# Local Well-Posedness of the Periodic Nonlinear Schrödinger Equation with a Quadratic Nonlinearity $\overline{u}^2$ in Negative Sobolev Spaces

**DOI:** 10.1007/s10884-023-10295-x

**Published:** 2023-08-07

**Authors:** Ruoyuan Liu

**Affiliations:** School of Mathematics, The University of Edinburgh and The Maxwell Institute for the Mathematical Sciences, James Clerk Maxwell Building, The King’s Buildings, Peter Guthrie Tait Road, Edinburgh, EH9 3FD UK

**Keywords:** Nonlinear Schrödinger equation, Well-posedness, 35Q55

## Abstract

We study low regularity local well-posedness of the nonlinear Schrödinger equation (NLS) with the quadratic nonlinearity $\overline{u}^2$, posed on one-dimensional and two-dimensional tori. While the relevant bilinear estimate with respect to the $X^{s, b}$-space is known to fail when the regularity *s* is below some threshold value, we establish local well-posedness for such low regularity by introducing modifications on the $X^{s, b}$-space.

## Introduction

### Quadratic Nonlinear Schrödinger Equations

In this paper, we consider the following Cauchy problem for the quadratic nonlinear Schrödinger equation (NLS) on periodic domains:

1.1 $$\begin{aligned} {\left\{ \begin{array}{ll} i \partial _tu + \Delta u = \overline{u}^2 \\ u|_{t = 0} = u_0 \end{array}\right. } \quad (x, t) \in \mathcal {M} \times \mathbb {R}, \end{aligned}$$

where $\mathcal {M} = \mathbb {T}$ or $\mathbb {T}^2$ with $\mathbb {T}= \mathbb {R}/ 2 \pi \mathbb {Z}$.

Our main goal is to establish low regularity local well-posedness of the quadratic NLS (1.1) on periodic domains $\mathbb {T}$ or $\mathbb {T}^2$. For instructive purposes, we first provide some background on the quadratic NLS

1.2 $$\begin{aligned} i \partial _tu + \Delta u = \mathcal {N} (u, u), \end{aligned}$$

where $\mathcal {N} (u, u)$ can be $u^2$, $\overline{u}^2$, or $|u|^2$. Note that on $\mathbb {R}^d$, if *u* is a solution to (1.2), then $u_\lambda (x, t):= \lambda ^2 u (\lambda x, \lambda ^2 t)$ is also a solution to (1.2) for any $\lambda > 0$. This scaling symmetry induces the following scaling critical Sobolev regularity:

$$\begin{aligned} s_{\text {crit}} = \frac{d}{2} - 2. \end{aligned}$$

When $d \le 3$, the scaling critical regularity is negative, which often fails to predict well-posedness and ill-posedness issues. In this paper, we mainly focus on the cases when $d = 1$ and $d = 2$.

Let us now review some previous results on the quadratic NLS (1.2), starting with the real line case. In [16], Kenig-Ponce-Vega used the Bourgain space $X^{s, b}$ (see Sect. 2.2) to prove local well-posedness of (1.2) on $\mathbb {R}$ for all types of nonlinearities $u^2$, $\overline{u}^2$, and $|u|^2$. Specifically, they established the following bilinear estimates:

1.3 $$\begin{aligned} \Vert u v \Vert _{X^{s, b - 1}}&\le \Vert u \Vert _{X^{s, b}} \Vert v \Vert _{X^{s, b}}, \end{aligned}$$

1.4 $$\begin{aligned} \Vert \overline{u} \overline{v} \Vert _{X^{s, b - 1}}&\le \Vert u \Vert _{X^{s, b}} \Vert v \Vert _{X^{s, b}} \end{aligned}$$

for $s > -\frac{3}{4}$ and $b = \frac{1}{2} +$ and^1^

1.5 $$\begin{aligned} \Vert u \overline{v} \Vert _{X^{s, b - 1}}&\le \Vert u \Vert _{X^{s, b}} \Vert v \Vert _{X^{s, b}} \end{aligned}$$

for $s > -\frac{1}{4}$ and $b = \frac{1}{2} +$. In addition, in the same paper, they showed that (1.3) and (1.4) fail for $s < -\frac{3}{4}$ and (1.5) fails for $s < -\frac{1}{4}$. The failure of these bilinear estimates at the endpoint regularities were established in [25]. Despite the failure of the bilinear estimate (1.3), Bejenaru-Tao [2] showed local well-posedness of (1.2) on $\mathbb {R}$ with nonlinearity $\mathcal {N} (u, u) = u^2$ for $s \ge -1$ by introducing weighted function spaces. Moreover, they proved ill-posedness of the same equation for $s < -1$. Later, Kishimoto [17] proved local well-posedness of (1.2) on $\mathbb {R}$ with $\mathcal {N} (u, u) = \overline{u}^2$ for $s \ge -1$ using different weighted function spaces. He also proved ill-posedness of the same equation for $s < -1$. Regarding (1.2) on $\mathbb {R}$ with $\mathcal {N} (u, u) = |u|^2$, Kishimoto [18] showed local well-posedness for $s \ge -\frac{1}{4}$ and ill-posedness for $s < -\frac{1}{4}$ (see also [22]). See also [13, 14, 21] for stronger ill-posedness results in the same ranges of *s*. For convenience, we summarize these results in Table 1. Note that for all these nonlinearities $u^2$, $\overline{u}^2$, and $|u|^2$, well-posedness and ill-posedness results are sharp. Also, for all these nonlinearities, ill-posedness occurs before *s* reaches the scaling critical regularity of (1.2) on $\mathbb {R}$: $s_{\text {crit}} = -\frac{3}{2}$.

**Table 1:** Known results for the quadratic NLS on $\mathbb {R}$ and $\mathbb {R}^2$

Setting	$\mathbb {R}$			$\mathbb {R}^2$		
Nonlinearity $\mathcal {N} (u, u)$	$u^2$	$\overline{u}^2$	$|u|^2$	$u^2$	$\overline{u}^2$	$|u|^2$
Scaling critical regularity	$s_{\text {crit}} = - \frac{3}{2}$			$s_{\text {crit}} = - 1$		
$X^{s, b}$-bilinear estimate	$s > - \frac{3}{4}$	$s > - \frac{3}{4}$	$s > - \frac{1}{4}$	$s > -\frac{3}{4}$	$s > - \frac{3}{4}$	$s > -\frac{1}{4}$
Failure of $X^{s, b}$-bilinear estimate	$s \le - \frac{3}{4}$	$s \le - \frac{3}{4}$	$s \le - \frac{1}{4}$	$s \le - \frac{3}{4}$	$s \le - \frac{3}{4}$	$s \le - \frac{1}{4}$
Local well-posedness	$s \ge -1$	$s \ge -1$	$s \ge - \frac{1}{4}$	$s > -1$	$s > -1$	$s \ge - \frac{1}{4}$
Ill-posedness	$s < -1$	$s < -1$	$s < -\frac{1}{4}$	$s \le -1$	$s \le -1$	$s < -\frac{1}{4}$

Note that in all cases, local well-posedness and ill-posedness results are sharp

Let us also mention well-posedness and ill-posedness results of (1.2) on $\mathbb {R}^2$, which are again summarized in Table 1. The $X^{s, b}$-bilinear estimates (1.3), (1.4), and (1.5) were established in [5, 30]. The failure of these $X^{s, b}$-bilinear estimates for lower values of *s* was shown in [5, 25]. For local well-posedness of (1.2) on $\mathbb {R}^2$, see [1, 18, 21]. For ill-posedness of (1.2) on $\mathbb {R}^2$, see [13, 14, 21]. From Table 1, we note that the ill-posedness on $\mathbb {R}^2$ for $\mathcal {N} (u, u) = |u|^2$ occurs before *s* reaches the scaling critical regularity $s_{\text {crit}} = -1$. Also, we can see that all well-posedness and ill-posedness results are sharp on $\mathbb {R}^2$.

We now turn our attention to well-posedness and ill-posedness results of (1.2) on periodic domains $\mathbb {T}$ and $\mathbb {T}^2$. The results are summarized in Table 2. On $\mathbb {T}$, for all nonlinearities $u^2$, $\overline{u}^2$, and $|u|^2$, the $X^{s, b}$-bilinear estimates (1.3), (1.4), and (1.5) for $s \ge 0$ follows immediately from the $L^3$-Strichartz estimate, which is obtained by interpolating the $L^4$-Strichartz estimate on $\mathbb {T}$ (see [3, 32]) and the trivial $L^2$-bound. In [16], Kenig-Ponce-Vega established bilinear estimates (1.3) (for $u^2$) and (1.4) (for $\overline{u}^2$) on $\mathbb {T}$ for $s > -\frac{1}{2}$ and $b = \frac{1}{2} +$ and showed corresponding local well-posedness results. They also showed that (1.3) and (1.4) fail on $\mathbb {T}$ when $s < -\frac{1}{2}$ and (1.5) (for $|u|^2$) fails on $\mathbb {T}$ when $s < 0$. Later, Kishimoto [21] showed ill-posedness of (1.2) on $\mathbb {T}$ with all types of nonlinearities for regularity ranges shown in Table 2. Here, we note that there are gaps between local well-posedness and ill-posedness results for nonlinearities $u^2$ and $\overline{u}^2$. Also, the quadratic NLS (1.2) with nonlinearity $|u|^2$ behaves worse on $\mathbb {T}$ than on $\mathbb {R}$, since ill-posedness on $\mathbb {T}$ occurs for a wider range of *s* than on $\mathbb {R}$.

For (1.2) on $\mathbb {T}^2$ with all nonlinearities $u^2$, $\overline{u}^2$, and $|u|^2$, the $X^{s, b}$-bilinear estimates (1.3), (1.4), and (1.5) for $s > 0$ follows from the $L^3$-Strichartz estimate with an $\varepsilon$ derivative loss, which is obtained by interpolating the $L^4$-Strichartz estimate on $\mathbb {T}^2$ (see Lemma 2.4) and the trivial $L^2$-bound. In [9], Grünrock showed the bilinear estimate (1.4) (for $\overline{u}^2$) for $s > -\frac{1}{2}$ and proved the corresponding local well-posedness result. In the same paper, he showed the failure of (1.3) (for $u^2$) on $\mathbb {T}^2$ when $s < 0$ and the failure of (1.4) (for $\overline{u}^2$) on $\mathbb {T}^2$ when $s < -\frac{1}{2}$. In [21], Kishimoto showed ill-posedness of (1.2) on $\mathbb {T}^2$ with all types of nonlinearities for regularity ranges shown in Table 2. In a recent work, Oh and the author [23] proved local well-posedness of (1.2) with nonlinearities $u^2$ and $|u|^2$ for $s = 0$ by establishing correponding $X^{s, b}$-bilinear estimates.

**Table 2:** Currect results for the quadratic NLS on $\mathbb {T}$ and $\mathbb {T}^2$

Setting	$\mathbb {T}$			$\mathbb {T}^2$		
Nonlinearity $\mathcal {N} (u, u)$	$u^2$	$\overline{u}^2$	$|u|^2$	$u^2$	$\overline{u}^2$	$|u|^2$
Scaling critical regularity	$s_{\text {crit}} = - \frac{3}{2}$			$s_{\text {crit}} = - 1$		
$X^{s, b}$-bilinear estimate	$s > - \frac{1}{2}$	$s > - \frac{1}{2}$	$s \ge 0$	$s \ge 0$	$s > - \frac{1}{2}$	$s \ge 0$
Failure of $X^{s, b}$-bilinear estimate	$s < - \frac{1}{2}$	$s < - \frac{1}{2}$	$s < 0$	$s < 0$	$s < - \frac{1}{2}$	$s < 0$
Local well-posedness	$s > - \frac{1}{2}$	$\pmb {s > - \frac{2}{3}}$	$s \ge 0$	$s \ge 0$	$\pmb {s > - \frac{2}{3}}$	$s \ge 0$
Ill-posedness	$s < -1$	$s < -1$	$s < 0$	$s \le -1$	$s \le -1$	$s < 0$

The boldface texts in the table refer to the results of this paper. Note that for the nonlinearity $|u|^2$, local well-posedness and ill-posedness results are sharp on both $\mathbb {T}$ and $\mathbb {T}^2$. For nonlinearities $u^2$ and $\overline{u}^2$ on either $\mathbb {T}$ or $\mathbb {T}^2$, there are gaps between local well-posedness and ill-posedness results

The long-time behaviors of the quadratic NLS (1.2) have also been studied. For global existence and scattering results, see [7, 8, 11, 15, 24, 28]. For nonexistence of non-trivial scattering solutions, see [27, 29]. For finite-time blowup results, see [12, 26].

As can be seen from Table 2, local well-posedness for the quadratic NLS with nonlinearity $|u|^2$ is complete, whereas for nonlinearities $u^2$ and $\overline{u}^2$, there are gaps between local well-posedness and ill-posedness results. The difference of well-posedness behaviors of these three nonlinearities is closed related to their distinct phase functions. By letting $n_1, n_2$ be the frequencies of the nonlinearity and *n* be the frequency of the duality term, we can write out the frequency interactions and phase functions for these three nonlinearities as in Table 3.

When the phase function is large, we expect some gain of regularities. For example, for nonlinearity $\overline{u}^2$ on $\mathbb {T}^2$, the phase function $|n|^2 + |n_1|^2 + |n_2|^2$ provides gain of derivatives, so that one can establish local well-posedness for nonlinearity $\overline{u}^2$ with very rough initial data. On the other hand, for nonlinearity $u^2$ on $\mathbb {T}^2$, the phase function $|n|^2 - |n_1|^2 - |n_2|^2 = 2 n_1 \cdot n_2$ can be very small if $n_1$ and $n_2$ are almost perpendicular to each other, so that local well-posedness with rough initial data is much harder. In this paper, we focus on shrinking the well-posedness gap for nonlinearity $\overline{u}^2$ by establishing local well-posedness with lower regularity. We also discuss some well-posedness issues for nonlinearity $u^2$ in Remark 1.6 below.

**Table 3:** Frequency interactions and phase functions for the quadratic NLS with nonlinearities $u^2$, $\overline{u}^2$, and $|u|^2$

Nonlinearity $\mathcal {N} (u, u)$	$u^2$	$\overline{u}^2$	$|u|^2$
Frequency interaction	$n - n_1 - n_2 = 0$	$n + n_1 + n_2 = 0$	$n - n_1 + n_2 = 0$
Phase function	$|n|^2 - |n_1|^2 - |n_2|^2$	$|n|^2 + |n_1|^2 + |n_2|^2$	$|n|^2 - |n_1|^2 + |n_2|^2$

We now look back on low regularity local well-posedness of the quadratic NLS (1.1) on $\mathbb {T}$ and $\mathbb {T}^2$. In this paper, we prove the following theorem.

#### Theorem 1.1

Let $\mathcal {M} = \mathbb {T}$ or $\mathbb {T}^2$. Then, the quadratic NLS (1.1) is locally well-posed in $H^s (\mathcal {M})$ for $s > -\frac{2}{3}$. More precisely, given any $u_0 \in H^s (\mathcal {M})$, there exists $T = T(\Vert u_0 \Vert _{H^s}) > 0$ and a unique solution $u \in C([-T, T]; H^s (\mathcal {M}))$ to (1.1) with $u|_{t = 0} = u_0$, and the solution *u* depends continuously on the initial data $u_0$.

Since local well-posedness of (1.1) for $s > -\frac{1}{2}$ was already shown in [16] on $\mathbb {T}$ and in [9] on $\mathbb {T}^2$, we mainly focus on the situation when $-\frac{2}{3} < s \le -\frac{1}{2}$. Our proof of Theorem 1.1 relies on modified $X^{s,b}$-spaces for the solutions, and so the uniqueness in the above statement holds only in the relevant function space (see the $Z^{s, b}$-norm in (2.4) and its local-in-time version in (2.6)). For the proof of Theorem 1.1, we will mainly focus on the case $\mathcal {M} = \mathbb {T}^2$ (see Remark 1.2). The idea of the proof of Theorem 1.1 is to introduce modifications on the $X^{s,b}$-space which enable us to prove the corresponding bilinear estimate. See Sect. 1.2 for more discussion on it.

Theorem 1.1 improves the previous local well-posedness results in [9, 16]. In addition, to the best of the author’s knowledge, these are the first local well-posedness results for the quadratic NLS on periodic domains below the regularity thresholds where the usual $X^{s, b}$-bilinear estimates fail. We also remark that the bound $s > -\frac{2}{3}$ is sharp (up to the endpoint regularity $s = -\frac{2}{3}$) in our approach. See Sect. 1.2 for more details.

#### Remark 1.2

In Theorem 1.1, the proof for $\mathcal {M} = \mathbb {T}$ follows from the proof for $\mathcal {M} = \mathbb {T}^2$ with minor modifications. Thus, in proving Theorem 1.1, we mainly restrict our attention on the case $\mathcal {M} = \mathbb {T}^2$.

### Modified Function Spaces

In this subsection, we briefly explain our strategy for proving Theorem 1.1.

In [2], Bejenaru-Tao reduced the well-posedness problem of the quadratic NLS (1.2) in $H^s (\mathbb {R}^d)$ or $H^s (\mathbb {T}^d)$ to finding a space-time norm $\Vert \cdot \Vert _{W^s}$ that satisfy the following properties^2^:

(i) (Monotonicity) If $|\widehat{f}| \le |\widehat{g}|$ pointwise, then

1.6 $$\begin{aligned} \Vert f \Vert _{W^s} \le \Vert g \Vert _{W^s}. \end{aligned}$$

Here, $\widehat{f}$ is the space-time Fourier transform of *f*.

(ii) ($H^s$-energy estimate) The following inequality holds:

1.7 $$\begin{aligned} \big \Vert \langle \xi \rangle ^s \widehat{f} (\xi , \tau ) \big \Vert _{L_\xi ^2 L_\tau ^1} \le \Vert f \Vert _{W^s}, \end{aligned}$$

where $$\langle \cdot \rangle = (1 + |\cdot |^2)^{\frac{1}{2}}$$.

(iii) (Homogeneous linear estimate) There exists $b \in \mathbb {R}$ such that

1.8 $$\begin{aligned} \Vert f \Vert _{W^s} \le \Vert f \Vert _{X^{s, b}}, \end{aligned}$$

where the $X^{s, b}$-norm is as defined in (2.1).

(iv) (Bilinear estimate) The following inequality holds:

1.9 $$\begin{aligned} \big \Vert \langle \tau + |\xi |^2 \rangle ^{-1} \mathcal {B} (\widehat{f}, \widehat{g}) \big \Vert _{\widehat{W}^s} \le \Vert f \Vert _{W^s} \Vert g \Vert _{W^s}, \end{aligned}$$

where $\widehat{W}^s$ is the same norm $W^s$ on the Fourier side and $\mathcal {B} (f, g)$ is equal to $f * g$ (if $\mathcal {N} (u, u) = u^2$), $\overline{\widetilde{f}} * \overline{\widetilde{g}}$ (if $\mathcal {N} (u, u) = \overline{u}^2$), or $f * \overline{\widetilde{g}}$ (if $\mathcal {N} (u, u) = |u|^2$). Here, $\widetilde{f} (\xi , \tau ) = f (- \xi , -\tau )$.

Now the task is to find suitable function spaces that satisfy the properties listed above. From now on, we restrict our attention to the nonlinearity $\mathcal {N} (u, u) = \overline{u}^2$ and the domain $\mathbb {T}^2$. As we have seen in the previous subsection, the usual $X^{s, b}$-bilinear estimate fails when the regularity is *very* low. This failure is caused by certain “dangerous” interactions. Thus, we need to introduce modifications on the $X^{s, b}$-space in order to reduce the effect by those “dangerous” interactions. In the following, we discuss several examples of such interactions and our strategy to deal with them.

#### Example 1

For a large number $N \in {\mathbb {N}}$, let

$$\begin{aligned} \widehat{u_N} (n, \tau )&= {\textbf{1}}_{\{ n = N e_1 \}} {\textbf{1}}_{[-1, 1]} (\tau + N^2), \\ \widehat{v_N} (n, \tau )&= {\textbf{1}}_{\{ n = - N e_1 \}} {\textbf{1}}_{[-1, 1]} (\tau + N^2), \end{aligned}$$

where $e_1 = (1, 0)$. Note that $\Vert u_N \Vert _{X^{s, b}} \sim N^s$ and $\Vert v_N \Vert _{X^{s, b}} \sim N^s$. A direct computation yields

$$\begin{aligned} \widehat{\overline{u_N} \overline{v_N}} (n, \tau ) \ge {\textbf{1}}_{\{n = 0\}} {\textbf{1}}_{[-1, 1]} (- \tau + 2N^2), \end{aligned}$$

and so $\Vert \overline{u_N} \overline{v_N} \Vert _{X^{s, b - 1}} \ge N^{2b - 2}$. Thus, the bilinear estimate (1.4) holds only if $2b - 2 \le 2\,s$ or $s \ge b - 1$. Since we need $b > \tfrac{1}{2}$, we require that $s > -\tfrac{1}{2}$.

In the above example, the frequency interaction is “high-high to low” and the modulation interaction is “low-low to high”. However, the modulation for $\widehat{\overline{u_N} \overline{v_N}}$ is not high enough for the desired $X^{s,b}$-bilinear estimate when $s \le -\tfrac{1}{2}$. To control the above interaction when $s \le - \tfrac{1}{2}$, we consider the following $Y^{s, b}$-norm introduced by Kishimoto [19]:

$$\begin{aligned} \Vert u \Vert _{Y^{s, b}} := \big \Vert \langle n \rangle ^s \widehat{u} (n, \tau ) \big \Vert _{\ell _n^2 L_\tau ^1 (\mathbb {Z}^2 \times \mathbb {R})} + \big \Vert \langle \tau + |n|^2 \rangle ^{\frac{s}{2} + b} \widehat{u} (n, \tau ) \big \Vert _{\ell _n^2 L_\tau ^2 (\mathbb {Z}^2 \times \mathbb {R})}, \end{aligned}$$

and we define the space $Z^{s, b} = X^{s, b} + Y^{s, b}$ via the norm

$$\begin{aligned} \Vert u \Vert _{Z^{s, b}} := \inf \{ \Vert u_1 \Vert _{X^{s, b}} + \Vert u_2 \Vert _{Y^{s, b}}: u_1 + u_2 = u \}. \end{aligned}$$

The $\ell _n^2 L_\tau ^1$-term in the $Y^{s, b}$-norm is needed to ensure that the $Z^{s, b}$-norm satisfies the $H^s$-energy estimate (1.7). It is not hard to check that the $Z^{s, b}$-norm satisfies the monotonicity property (1.6), the $H^s$-energy estimate (1.7), and the homogeneous linear estimate (1.8). Note that for $s \le 0$ and $b > \frac{1}{2}$, if $\mathop {\textrm{supp}}\limits \widehat{u} \subset \{ |\tau + |n|^2| \le |n|^2 \}$, then we have

$$\begin{aligned} \Vert u \Vert _{Z^{s, b}} \sim \Vert u \Vert _{X^{s, b}} \le \Vert u \Vert _{Y^{s, b}}; \end{aligned}$$

if $\mathop {\textrm{supp}}\limits \widehat{u} \subset \{ |\tau + |n|^2| \ge |n|^2 \}$, then we have

$$\begin{aligned} \Vert u \Vert _{Z^{s, b}} \sim \Vert u \Vert _{Y^{s, b}} \le \Vert u \Vert _{X^{s, b}}. \end{aligned}$$

In Sect. 2.3, we will revisit this $Z^{s, b}$-norm, which will be defined in a more precise manner for practical purposes.

In Example 1, because of the high modulation of $\widehat{\overline{u_N} \overline{v_N}}$, the $\widehat{Z}^{s, b}$-norm (i.e. the $Z^{s, b}$-norm on the Fourier side) of $\langle \tau + |n|^2 \rangle ^{-1} \widehat{\overline{u_N} \overline{v_N}}$ is small enough to obtain the desired bilinear estimate (1.9) for $s \le - \frac{1}{2}$. One can easily check that using the $Z^{s, b}$-norm, the bilinear estimate for the above example holds for $s \ge 2b - 2$. This is better than $s > -\tfrac{1}{2}$ as long as $\tfrac{1}{2} < b \le \tfrac{3}{4}$.

Let us take a look at another example using the $Z^{s, b}$-norm assuming that $s \le 0$.

#### Example 2

For a large number $N \in {\mathbb {N}}$, let

$$\begin{aligned} \widehat{u_N} (n, \tau )&= {\textbf{1}}_{\{n = N e_1\}} {\textbf{1}}_{[-1, 1]} (\tau + N^2), \\ \widehat{v_N} (n, \tau )&= {\textbf{1}}_{\{n = - N e_1\}} {\textbf{1}}_{[-1, 1]} (-\tau + N^2). \end{aligned}$$

A direct computation yields

$$\begin{aligned} \widehat{\overline{u_N} \overline{v_N}} (n, \tau ) \ge {\textbf{1}}_{\{n = 0\}} {\textbf{1}}_{[-1, 1]} (\tau ). \end{aligned}$$

Note that in this example, the frequency interaction is “high-high to low” and the modulation interaction is “low-high to low”. We can compute their corresponding $Z^{s,b}$-norms as follows:

$$\begin{aligned}&\Vert u_N \Vert _{Z^{s, b}} \sim \Vert u_N \Vert _{X^{s, b}} \sim N^s \\&\Vert v_N \Vert _{Z^{s, b}} \sim \Vert v_N \Vert _{Y^{s, b}} \sim N^{s + 2b} \\&\big \Vert \langle \tau + |n|^2 \rangle ^{-1} \widehat{\overline{u_N} \overline{v_N}} \big \Vert _{\widehat{Z}^{s, b}} \ge 1. \end{aligned}$$

Thus, the bilinear estimate (1.9) with $W = Z^{s, b}$ holds only if $0 \le 2\,s + 2b$ or $s \ge -b$.

Combining Example 1 and Example 2, we notice that the regularity *s* needs to satisfy $s \ge 2b - 2$ and $s \ge -b$. These two lower bounds become optimal when $b = \frac{2}{3}$, so that $s = - \frac{2}{3}$ seems to be the threshold of the bilinear estimate (1.9) with $W^s = Z^{s, b}$. In fact, we will show in Sect. 3 that the bilinear estimate (1.9) with $W^s = Z^{s, \frac{2}{3}}$ holds when $s > - \frac{2}{3}$ (see Remark 1.4 for a discussion on the slight loss of regularity). See Sect. 3 for more details.

We conclude this introduction by stating several remarks.

#### Remark 1.3

On $\mathbb {T}^d$, it is possible to use a scaling argument to prove local well-posedness for large initial data given that one can first obtain small data local well-posedness. See [6]. However, we do not pursue the scaling argument in this paper and instead rely on the time localization (Lemma 2.3) to prove local well-posedness for large initial data.

#### Remark 1.4

In [2, 17], a Besov refinement was considered in constructing desired function spaces so that the endpoint regularity (i.e. $s = -1$ for the quadratic NLS (1.2) on $\mathbb {R}$ with $\mathcal {N}(u, u) = u^2$ or $\overline{u}^2$) can be handled. Similar Besov refinements were used by [10, 20] in the context of the Korteweg-de Vris equation.

For the quadratic NLS (1.1) on $\mathbb {T}^2$, however, such Besov modification does not seem to be enough to cover the case when $s = - \frac{2}{3}$. This is mainly due to the fact that our approach relies heavily on the $L^4$-Strichartz estimate on $\mathbb {T}^2$ (see Lemma 2.4), which has an $\varepsilon$ loss of derivative.

For the quadratic NLS (1.1) on $\mathbb {T}$, since the $L^4$-Strichartz estimate on $\mathbb {T}$ (see [3]) does not have any derivative loss, it seems possible to adapt the Besov modification to our estimate so that the endpoint case can be included.

#### Remark 1.5

For the quadratic NLS (1.1) on $\mathbb {T}$ and $\mathbb {T}^2$, there are still gaps between local well-posedness and ill-posedness results (see Table 2). Specifically, on $\mathbb {T}$, well-posedness issues of (1.1) for $-1 \le s \le - \frac{2}{3}$ remain open; on $\mathbb {T}^2$, well-posedness issues of (1.1) for $-1 < s \le - \frac{2}{3}$ remain open. One possible strategy for improving our local well-posedness arguments is to introduce weighted spaces as in [1, 2, 17, 19] in the context of Euclidean spaces.

#### Remark 1.6

Let us consider the quadratic NLS (1.2) with $\mathcal {N} (u, u) = u^2$. On $\mathbb {T}$, local well-posedness is known to hold for $s > -\frac{1}{2}$ and ill-posedness holds for $s < -1$. We believe that the method of using modified function spaces should be able to produce better local well-posedness results, but one may need to use the weighted spaces as in [1, 2] to handle the corresponding bilinear estimate.

For the quadratic NLS (1.2) with $\mathcal {N} (u, u) = u^2$ on $\mathbb {T}^2$, local well-posedness is known to hold for $s \ge 0$ and ill-posedness holds for $s \le -1$. However, it seems unlikely that the method of finding modified function spaces as illustrated at the beginning of Sect. 1.2 works in the range $s < 0$. This is due to the following example in [9]. For a large number $N \in \mathbb {N}$, let

$$\begin{aligned} \widehat{u_N} (n, \tau ) = {\textbf{1}}_{\{n = N e_1\}} {\textbf{1}}_{[-1, 1]} (\tau + N^2), \\ \widehat{v_N} (n, \tau ) = {\textbf{1}}_{\{n = N e_2\}} {\textbf{1}}_{[-1, 1]} (\tau + N^2), \end{aligned}$$

where $e_1 = (1, 0)$ and $e_2 = (0, 1)$. A direct computation yields

$$\begin{aligned} \widehat{u_N v_N} (n, \tau )&= {\textbf{1}}_{\{n = N (e_1 + e_2) \}} \max \big \{ 0, \min \{ 2 - \tau - 2 N^2, 2 + \tau + 2 N^2 \} \big \} \\&\ge {\textbf{1}}_{\{n = N (e_1 + e_2)\}} {\textbf{1}}_{[-1, 1]} (\tau + 2N^2). \end{aligned}$$

In this example, the frequency interaction is “high-high to high” and the modulation interaction is “low-low to low”, which means that there seems to be no way to utilize the modulation to improve the bilinear estimate. Note that this “low-low to low” interaction does not occur for the nonlinearity $\mathcal {N} (u, u) = \overline{u}^2$, which can be seen from the computations at the beginning of Subcase 2.3 of Lemma 3.2 below and Case 3 of Lemma 3.3 below.

For any $s \in \mathbb {R}$ and $b \in \mathbb {R}$, we have

$$\begin{aligned} \Vert u_N \Vert _{X^{s, b}} \sim \Vert v_N \Vert _{X^{s, b}} \sim \Vert \langle \tau + |n|^2 \rangle ^{-1} \widehat{u_N v_N} \Vert _{\widehat{X}^{s, b}} \sim N^s, \end{aligned}$$

where the $\widehat{X}^{s, b}$-norm is the $X^{s, b}$-norm on the Fourier side. Thus, we observe that due to the homogeneous linear estimate (1.8) and the similar structures of $\widehat{u_N}$, $\widehat{v_N}$, and $\widehat{u_N v_N}$, any qualified modified norm $\Vert \cdot \Vert _{W^s}$ should decrease the corresponding norms of $\widehat{u_N}$, $\widehat{v_N}$, and $\langle \tau + |n|^2 \rangle ^{-1} \widehat{u_N v_N}$ with the same rate (with respect to *N*). Suppose that there exists $a \ge 0$ such that

$$\begin{aligned} \Vert u_N \Vert _{W^s} \sim \Vert v_N \Vert _{W^s} \sim \Vert \langle \tau + |n|^2 \rangle ^{-1} \widehat{u_N v_N} \Vert _{\widehat{W}^s} \sim N^{s - a}. \end{aligned}$$

where the $\widehat{W}^s$-norm is the $W^s$-norm on the Fourier side. Then, for the bilinear estimate (1.9) to hold, we must have

$$\begin{aligned} N^{s - a} \le N^{2s - 2a}, \end{aligned}$$

so that $s - a \ge 0$ or $s \ge a \ge 0$. Therefore, we do not expect that the method of finding the $W^s$-norm for proving local well-posedness works for the quadratic NLS (1.2) with $\mathcal {N} (u, u) = u^2$ on $\mathbb {T}^2$ for $s < 0$, and it is possible that some ill-posedness results may hold in this range.

## Notations and Function Spaces

In this section, we introduce some notations and function spaces that enable us to prove local well-posedness of (1.1) in low regularity settings.

### Notations

Throughout this paper, we drop the inessential factor of $2\pi$. For a space-time distribution *u*, we write $\widehat{u}$ or $\mathcal {F}_{x, t} u$ to denote the space-time Fourier transform of *u*. If a function $\phi$ only has a space (or time) variable, then we use $\widehat{\phi }$ to denote the Fourier transform of $\phi$ with respect to the space (or time, respectively) variable. For any function *f*, the function $\widetilde{f}$ is the reflection of *f*, i.e. $\widetilde{f}(x) = f(-x)$. We also set $\langle \,\cdot \, \rangle = (1 + |\cdot |^2)^\frac{1}{2}$.

We use $A \le B$ to denote $A \le CB$ for some constant $C > 0$. We write $A \sim B$ if we have $A \le B$ and $B \le A$. We may use subscripts to denote dependence on external parameters. We also use $a +$ and $a -$ to denote $a + \varepsilon$ and $a - \varepsilon$, respectively, for sufficiently small $\varepsilon > 0$.

Given a dyadic number $N \in 2^{\mathbb {N}\cup \{0\}}$, if $N \ge 2$, we let $P_N$ be the spatial frequency projector onto the frequencies

$$\begin{aligned} \mathfrak {P}_N := \{ (n, \tau ) \in \mathbb {Z}^2 \times \mathbb {R}: \tfrac{1}{2} N < |n| \le N \}. \end{aligned}$$

If $N = 1$, we let $P_1$ be the spatial frequency projector onto the frequencies

$$\begin{aligned} \mathfrak {P}_1 := \{ (n, \tau ) \in \mathbb {Z}^2 \times \mathbb {R}: |n| \le 1 \}. \end{aligned}$$

For a space-time distribution *u*, we also write $u_N:= P_N u$ for simplicity.

### Fourier Restriction Norm Method

In this subsection, we recall the definition and estimates of $X^{s, b}$-spaces for the Schrödinger equations, which were first introduced by Bourgain [3]. Given $s, b \in \mathbb {R}$, we define the space $X^{s, b} = X^{s, b} (\mathbb {T}^2 \times \mathbb {R})$ to be the completion of functions that are smooth in space and Schwartz in time with respect to the following norm:

2.1 $$\begin{aligned} \Vert u \Vert _{X^{s, b}} := \big \Vert \langle n \rangle ^s \langle \tau + |n|^2 \rangle ^b \widehat{u} (n, \tau ) \big \Vert _{\ell _n^2 L_\tau ^2 (\mathbb {Z}^2 \times \mathbb {R})}. \end{aligned}$$

We now present and recall some estimates related to $X^{s, b}$-norms, starting with the following stronger version of the usual homogeneous linear estimate of the $X^{s, b}$-norm as in [3, 31].

#### Lemma 2.1

Let $\varphi$ be a smooth function supported on $[-2, 2]$. Let $s \in \mathbb {R}$, $b \le 1$, and $k \in \mathbb {N}\cup \{0\}$. Then, we have

2.2 $$\begin{aligned} \big \Vert t^k \varphi (t) e^{it \Delta } \phi \big \Vert _{X^{s, b}} \le _{\varphi } 3^k \Vert \phi \Vert _{H^s (\mathbb {T}^2)} \end{aligned}$$

#### Proof

Note that by the fact that $b \le 1$, we have

$$\begin{aligned} \big \Vert t^k \varphi (t) e^{it \Delta } \phi \big \Vert _{X^{s, b}}&= \Big \Vert \widehat{(\cdot )^k \varphi } (\tau + |n|^2) \langle \tau + |n|^2 \rangle ^b \langle n \rangle ^s \widehat{\phi }(n) \Big \Vert _{\ell _n^2 L_\tau ^2} \\&= \big \Vert t^k \varphi (t) \big \Vert _{H^b (\mathbb {R})} \Vert \phi \Vert _{H^s (\mathbb {T}^2)} \\&\le \big ( \big \Vert t^k \varphi (t) \big \Vert _{L^2 (\mathbb {R})} + \big \Vert \partial _t\big ( t^k \varphi (t) \big ) \big \Vert _{L^2 (\mathbb {R})} \big ) \Vert \phi \Vert _{H^s (\mathbb {T}^2)} \\&\le (2^k + k 2^{k - 1} ) (\Vert \varphi \Vert _{L^2 (\mathbb {R})} + \Vert \partial _t\varphi \Vert _{L^2 (\mathbb {R})}) \Vert \phi \Vert _{H^s (\mathbb {T}^2)} \\&\le _\varphi 3^k \Vert \phi \Vert _{H^s (\mathbb {T}^2)}, \end{aligned}$$

as desired. $$\square $$

#### Remark 2.2

In fact, the estimate (2.2) holds for all $b \in \mathbb {R}$. For the proof of our local well-posedness result, however, we will only need the estimate (2.2) for $b \le 1$.

Next, we recall the following time localization estimate. For a proof, see [3, 31].

#### Lemma 2.3

Let $s \in \mathbb {R}$, $-\frac{1}{2}< b_1 \le b_2 < \frac{1}{2}$, and $0 < T \le 1$. Let $\varphi$ be a Schwartz function and let $\varphi _T (t):= \varphi (t / T)$. Then, we have

$$\begin{aligned} \Vert \varphi _T u \Vert _{X^{s, b_1}} \le _\varphi T^{b_2 - b_1} \Vert u \Vert _{X^{s, b_2}}. \end{aligned}$$

We also record the following $L^4$-Strichartz estimate on $\mathbb {T}^2$. For a proof, see [3, 4].

#### Lemma 2.4

Let *N* be a dyadic number. Then, we have

$$\begin{aligned} \Vert u_N \Vert _{L_t^4([-1, 1]; L_x^4(\mathbb {T}^2))} \le N^{s} \Vert u_N \Vert _{X^{0, b}}, \end{aligned}$$

where $$0< s < \frac{1}{2}$$ and $$b > \frac{1 - s}{2}$$.

### Modified Function Spaces

In this subsection, we define our solution space for the quadratic NLS (1.1) in the low regularity setting and establish corresponding linear estimates.

Given $s, b \in \mathbb {R}$, we define the space $Y^{s, b} = Y^{s, b} (\mathbb {T}^2 \times \mathbb {R})$ to be the completion of functions that are smooth in space and Schwartz in time with respect to the norm

2.3 $$\begin{aligned} \begin{aligned} \Vert u \Vert _{Y^{s, b}}&:= \big \Vert \langle n \rangle ^s \widehat{u} (n, \tau ) \big \Vert _{\ell _n^2 L_\tau ^1 (\mathbb {Z}^2 \times \mathbb {R})} + \big \Vert \langle \tau + |n|^2 \rangle ^{\frac{s}{2} + b} \widehat{u} (n, \tau ) \big \Vert _{\ell _n^2 L_\tau ^2 (\mathbb {Z}^2 \times \mathbb {R})}. \end{aligned} \end{aligned}$$

The idea of this modification comes from Kishimoto [19].

We now define the space $Z^{s, b}$ via the norm

2.4 $$\begin{aligned} \Vert u \Vert _{Z^{s, b}} := \Vert P_{\text {lo}} u \Vert _{X^{s, b}} + \Vert P_{\text {hi}} u \Vert _{Y^{s, b}}, \end{aligned}$$

where $P_{\text {lo}}$ is the space-time frequency projector onto the frequencies $\{ |\tau + |n|^2| < 2^{-10} |n|^2 \}$ and $P_{\text {hi}}$ is the space-time frequency projector onto the frequencies $\{ |\tau + |n|^2| \ge 2^{-10} |n|^2 \}$. From the definition, we observe that the $Z^{s, b}$-norm has the monotonicity property: if $|\widehat{u_1}| \le |\widehat{u_2}|$ pointwise, then

2.5 $$\begin{aligned} \Vert u_1 \Vert _{Z^{s, b}} \le \Vert u_2 \Vert _{Z^{s, b}}. \end{aligned}$$

For $T > 0$, we define the space $Z_T^{s, b}$ as the restriction of the $Z^{s, b}$-space onto the time interval $[-T, T]$ via the norm:

2.6 $$\begin{aligned} \Vert u \Vert _{Z_T^{s, b}} := \inf \big \{ \Vert v \Vert _{Z^{s, b}} : v |_{[-T, T]} = u \big \}. \end{aligned}$$

Note that the $Z_T^{s, b}$-space is complete.

For convenience and conciseness, later on we may use the notations $\widehat{X}^{s, b}$, $\widehat{Y}^{s, b}$, and $\widehat{Z}^{s, b}$ to denote the corresponding norms on the Fourier side. In other words, for a complex-valued function *f* defined on $\mathbb {Z}^2 \times \mathbb {R}$, we write

$$\begin{aligned} \Vert f \Vert _{\widehat{X}^{s, b}}&= \Vert \mathcal {F}^{-1} (f) \Vert _{X^{s, b}}, \\ \Vert f \Vert _{\widehat{Y}^{s, b}}&= \Vert \mathcal {F}^{-1} (f) \Vert _{Y^{s, b}}, \\ \Vert f \Vert _{\widehat{Z}^{s, b}}&= \Vert \mathcal {F}^{-1} (f) \Vert _{Z^{s, b}}, \end{aligned}$$

where $\mathcal {F}^{-1}$ is the inverse Fourier transform.

We now establish some linear estimates of the $Z^{s, b}$-norm. We start with the following $H^s$-energy estimate.

#### Lemma 2.5

Let $s \in \mathbb {R}$ and $b > \frac{1}{2}$. Then, we have

$$\begin{aligned} \big \Vert \langle n \rangle ^s \widehat{u} (n, \tau ) \big \Vert _{\ell _n^2 L_\tau ^1} \le \Vert u \Vert _{Z^{s, b}}. \end{aligned}$$

#### Proof

By the definition of the $Z^{s, b}$-norm in (2.4), we know that it suffices to show the following two estimates:

2.7 $$\begin{aligned} \big \Vert \langle n \rangle ^s \widehat{u} (n, \tau ) \big \Vert _{\ell _n^2 L_\tau ^1}&\le \Vert u \Vert _{X^{s, b}}, \end{aligned}$$

2.8 $$\begin{aligned} \big \Vert \langle n \rangle ^s \widehat{u} (n, \tau ) \big \Vert _{\ell _n^2 L_\tau ^1}&\le \Vert u \Vert _{Y^{s, b}}. \end{aligned}$$

Since $b > \frac{1}{2}$, we use the Cauchy-Schwarz inequality in $\tau$ to obtain

$$\begin{aligned} \big \Vert \langle n \rangle ^s \widehat{u} (n, \tau ) \big \Vert _{\ell _n^2 L_\tau ^1} \le \big \Vert \langle n \rangle ^s \langle \tau + |n|^2 \rangle ^b \widehat{u} (n, \tau ) \big \Vert _{\ell _n^2 L_\tau ^2} \le \Vert u \Vert _{X^{s, b}}, \end{aligned}$$

so that we obtain (2.7). Also, note that (2.8) is easily obtained from the definition of the $Y^{s, b}$-norm in (2.3). $\square$

The above lemma implies the following embedding result.

#### Lemma 2.6

Let $s \in \mathbb {R}$, $b > \frac{1}{2}$, and $T > 0$. Then, we have

$$\begin{aligned} \Vert u \Vert _{C([-T, T]; H^s (\mathbb {T}^2))} \le \Vert u \Vert _{Z_T^{s, b}}. \end{aligned}$$

Consequently, the embedding

$$\begin{aligned} Z_T^{s, b} \hookrightarrow C([-T, T]; H^s (\mathbb {T}^2)) \end{aligned}$$

holds.

#### Proof

Let $\varepsilon > 0$ and let *v* be an extension of *u* outside of $[-T, T]$ such that

2.9 $$\begin{aligned} \Vert v \Vert _{Z^{s, b}} \le \Vert u \Vert _{Z_T^{s, b}} + \varepsilon . \end{aligned}$$

Note that we have the following embedding

2.10 $$\begin{aligned} \Vert v \Vert _{C([-T, T]; H^s (\mathbb {T}^2))} \le \big \Vert \langle n \rangle ^s \widehat{v} (n, \tau ) \big \Vert _{\ell _n^2 L_\tau ^1}. \end{aligned}$$

Thus, by (2.10), Lemma 2.5, and (2.9), we obtain

$$\begin{aligned} \Vert u \Vert _{C([-T, T]; H^s (\mathbb {T}^2))}&= \Vert v \Vert _{C([-T, T]; H^s (\mathbb {T}^2))} \\&\le \big \Vert \langle n \rangle ^s \widehat{v} (n, \tau ) \big \Vert _{\ell _n^2 L_\tau ^1} \\&\le \Vert v \Vert _{Z^{s, b}} \\&\le \Vert u \Vert _{Z_T^{s, b}} + \varepsilon , \end{aligned}$$

and so the desired estimate follows since $\varepsilon > 0$ can be arbitrarily small. $\square$

Lastly, we show the following lemma, which shows that the $X^{s, b}$-space is embedded in the $Z^{s, b}$-space.

#### Lemma 2.7

Let $s \le 0$ and $b > \frac{1}{2}$. Then, we have

$$\begin{aligned} \Vert u \Vert _{Z^{s, b}} \le \Vert u \Vert _{X^{s, b}}. \end{aligned}$$

#### Proof

We recall from (2.4) that

$$\begin{aligned} \Vert u \Vert _{Z^{s, b}} = \Vert P_{\text {lo}} u \Vert _{X^{s, b}} + \Vert P_{\text {hi}} u \Vert _{Y^{s, b}}, \end{aligned}$$

where $P_{lo }$ projects the space-time frequencies onto $\{ |\tau + |n|^2| < 2^{-10} |n|^2 \}$ and $P_{hi }$ projects the space-time frequencies onto $\{ |\tau + |n|^2| \ge 2^{-10} |n|^2 \}$. Note that we have

$$\begin{aligned} \Vert P_{\text {lo}} u \Vert _{X^{s, b}} \le \Vert u \Vert _{X^{s, b}}. \end{aligned}$$

For the $\Vert P_{\text {hi}} u \Vert _{Y^{s, b}}$ term, note that by the Cauchy-Schwarz inequality, we have

$$\begin{aligned} \big \Vert \langle n \rangle ^s \widehat{u} (n, \tau ) \big \Vert _{\ell _n^2 L_\tau ^1} \le \big \Vert \langle n \rangle ^s \langle \tau + |n|^2 \rangle ^{b} \widehat{u} (n, \tau ) \big \Vert _{\ell _n^2 L_\tau ^2} = \Vert u \Vert _{X^{s, b}}, \end{aligned}$$

since $b > \frac{1}{2}$. Also, we have

$$\begin{aligned} \big \Vert \langle \tau + |n|^2 \rangle ^{\frac{s}{2} + b} \widehat{u} (n, \tau ) {\textbf{1}}_{\{|\tau + |n|^2| \ge 2^{-10} |n|^2\}} \big \Vert _{\ell _n^2 L_\tau ^2} \le \big \Vert \langle n \rangle ^s \langle \tau + |n|^2 \rangle ^{b} \widehat{u} (n, \tau ) \big \Vert _{\ell _n^2 L_\tau ^2} = \Vert u \Vert _{X^{s, b}}. \end{aligned}$$

Thus, we obtain that $\Vert P_{\text {hi}} u \Vert _{Y^{s, b}} \le \Vert u \Vert _{X^{s, b}}$, so that we achieve the desired inequality. $\square$

## Bilinear Estimate

In this section, we establish the crucial bilinear estimate with respect to the $Z^{s, b}$-norm introduced in the previous section. Specifically, we show the following proposition.

### Proposition 3.1

Let $- \frac{2}{3} < s \le - \frac{1}{2}$ and $0 < T \le \frac{1}{2}$. Let $\varphi : \mathbb {R}\rightarrow [0, 1]$ be a smooth function such that $\varphi \equiv 1$ on $[-1, 1]$ and $\varphi \equiv 0$ outside of $[-2, 2]$, and let $\varphi _T (t):= \varphi (t / T)$. Then, we have

$$\begin{aligned} \big \Vert \langle \tau + |n|^2 \rangle ^{-1} \mathcal {F}_{x, t} \big ( \varphi _T \overline{u} \cdot \varphi _T \overline{v} \big ) (n, \tau ) \big \Vert _{\widehat{Z}^{s, \frac{2}{3}}} \le _\varphi T^\theta \Vert u \Vert _{Z^{s, \frac{2}{3}}} \Vert v \Vert _{Z^{s, \frac{2}{3}}} \end{aligned}$$

for some $$\theta > 0$$.

Let us first consider two particular cases of Proposition 3.1. We start with the following “high-low interaction” estimate.

### Lemma 3.2

Let $-\frac{2}{3} < s \le -\frac{1}{2}$ and $0 < T \le \frac{1}{2}$. Let *N*, $N_1$, and $N_2$ be dyadic numbers. Let $\varphi : \mathbb {R}\rightarrow [0, 1]$ be a smooth function such that $\varphi \equiv 1$ on $[-1, 1]$ and $\varphi \equiv 0$ outside of $[-2, 2]$, and let $\varphi _T (t):= \varphi (t / T)$.

(i) If $2^{-5} N \le N_1 \le 2^5 N$ and $N_2 \le 2^6 N$, we have

$$\begin{aligned} \big \Vert \langle \tau + |n|^2 \rangle ^{-1} \mathcal {F}_{x, t} \big ( \varphi _T \overline{u_{N_1}} \cdot \varphi _T \overline{v_{N_2}} \big ) (n, \tau ) \big \Vert _{\widehat{Z}^{s, \frac{2}{3}} (\mathfrak {P}_N)} \le _\varphi N_2^{-\delta } T^\theta \Vert u_{N_1} \Vert _{Z^{s, \frac{2}{3}}} \Vert v_{N_2} \Vert _{Z^{s, \frac{2}{3}}} \end{aligned}$$

for some $\delta > 0$ and $\theta > 0$.

(ii) If $2^{-5} N \le N_2 \le 2^5 N$ and $N_1 \le 2^6 N$, we have

$$\begin{aligned} \big \Vert \langle \tau + |n|^2 \rangle ^{-1} \mathcal {F}_{x, t} \big ( \varphi _T \overline{u_{N_1}} \cdot \varphi _T \overline{v_{N_2}} \big ) (n, \tau ) \big \Vert _{\widehat{Z}^{s, \frac{2}{3}} (\mathfrak {P}_N)} \le _\varphi N_1^{-\delta } T^\theta \Vert u_{N_1} \Vert _{Z^{s, \frac{2}{3}}} \Vert v_{N_2} \Vert _{Z^{s, \frac{2}{3}}} \end{aligned}$$

for some $\delta > 0$ and $\theta > 0$.

### Proof

By the symmetry of *u* and *v*, it suffices to prove (i). Below we use $(n_1, \tau _1)$ as the variables of $\widehat{ \varphi _T u_{N_1} }$ or $\widehat{u_{N_1}}$ and $(n_2, \tau _2)$ as the variables of $\widehat{ \varphi _T v_{N_2} }$ or $\widehat{v_{N_2}}$. Note that we have the relations $\tau + \tau _1 + \tau _2 = 0$ and $n + n_1 + n_2 = 0$. We also recall the notation $\widetilde{f} (x) = f(-x)$.

We divide the argument into two main cases depending on the relationship between the modulation function $\tau + |n|^2$ and $|n|^2$.

**Case 1** $$| \tau + |n|^2 | \ge 2^{-10} |n|^2$$.

In this case, we need to evaluate the $\mathcal {F}_{x, t} \big ( \varphi _T \overline{u_{N_1}} \cdot \varphi _T \overline{v_{N_2}} \big )$ term using the $\widehat{Y}^{s, \frac{2}{3}}$-norm, and we need to evaluate both the $\ell _n^2 L_\tau ^1$ term and the $\ell _n^2 L_\tau ^2$ term. We consider the following three subcases.

**Subcase 1.1** $$|\tau _1 + |n_1|^2| \ge 2^{-10} |n_1|^2$$.

In this subcase, we need to estimate $u_{N_1}$ using the $Y^{s, \frac{2}{3}}$-norm. By Young’s convolution inequality, Lemma 2.3, the Cauchy-Schwarz inequality, and Lemma 2.5, we obtain

3.1 $$\begin{aligned} \begin{aligned} \big \Vert&\langle \tau + |n|^2 \rangle ^{\frac{s}{2} - \frac{1}{3}} \mathcal {F}_{x, t} \big ( \varphi _T \overline{u_{N_1}} \cdot \varphi _T \overline{v_{N_2}} \big ) \big \Vert _{\ell _n^2 L_\tau ^2 (\mathfrak {P}_N)} \\&= \bigg \Vert \langle \tau + |n|^2 \rangle ^{\frac{s}{2} - \frac{1}{3}} \widetilde{\widehat{\varphi _T^2 u_{N_1}}} * \widetilde{\widehat{v_{N_2}}} \bigg \Vert _{\ell _n^2 L_\tau ^2 (\mathfrak {P}_N)} \\&\le N^{s - \frac{2}{3}} \Big \Vert \widehat{\varphi _T^2 u_{N_1}} \Big \Vert _{\ell _{n_1}^2 L_{\tau _1}^2} \big \Vert \widehat{v_{N_2}} \big \Vert _{\ell _{n_2}^1 L_{\tau _2}^1} \\&\le _\varphi N^{s - \frac{2}{3}} T^{\varepsilon } \big \Vert \langle \tau _1 + |n_1|^2 \rangle ^\varepsilon \widehat{u_{N_1}} \big \Vert _{\ell _{n_1}^2 L_{\tau _1}^2} N_2^{- s + 1} \big \Vert \langle n_2 \rangle ^s \widehat{v_{N_2}} \big \Vert _{\ell _{n_2}^2 L_{\tau _2}^1} \\&\le N^{s - \frac{2}{3}} T^\varepsilon N_1^{-s - \frac{4}{3} + 2\varepsilon } \Vert u_{N_1} \Vert _{Y^{s, \frac{2}{3}}} N_2^{- s + 1} \Vert v_{N_2} \Vert _{Z^{s, \frac{2}{3}}} \\&\le N^{-2 + 2\varepsilon } N_2^{-s + 1} T^\varepsilon \Vert u_{N_1} \Vert _{Z^{s, \frac{2}{3}}} \Vert v_{N_2} \Vert _{Z^{s, \frac{2}{3}}}, \end{aligned} \end{aligned}$$

where $$\varepsilon > 0$$ is arbitrarily small. Since $$ - s + 1 > 0$$ given $$s \le -\frac{1}{2}$$, the above estimate is acceptable if $$-s - 1 + 2\varepsilon < 0$$, which is valid given $$s > - \frac{2}{3}$$ and $$\varepsilon > 0$$ sufficiently small.

Also, by the Cauchy-Schwarz inequality, we get

$$\begin{aligned} \big \Vert&\langle n \rangle ^s \langle \tau + |n|^2 \rangle ^{- 1} \mathcal {F}_{x, t} \big ( \varphi _T \overline{u_{N_1}} \cdot \varphi _T \overline{v_{N_2}} \big ) \big \Vert _{\ell _n^2 L_\tau ^1 (\mathfrak {P}_N)} \\&\le \big \Vert \langle n \rangle ^s \langle \tau + |n|^2 \rangle ^{- \frac{1}{3}} \mathcal {F}_{x, t} \big ( \varphi _T \overline{u_{N_1}} \cdot \varphi _T \overline{v_{N_2}} \big ) \big \Vert _{\ell _n^2 L_\tau ^2 (\mathfrak {P}_N)}, \end{aligned}$$

which can be estimated similarly as in (3.1). Combining the above two estimates, we obtain the desired inequality.

**Subcase 1.2** $$|\tau _2 + |n_2|^2| \ge 2^{-10} |n_2|^2$$.

In this subcase, we need to estimate $v_{N_2}$ using the $Y^{s, \frac{2}{3}}$-norm. By Young’s convolution inequality, the Cauchy-Schwarz inequality, Lemma 2.3, and Lemma 2.5, we obtain

3.2 $$\begin{aligned} \begin{aligned} \big \Vert&\langle \tau + |n|^2 \rangle ^{\frac{s}{2} - \frac{1}{3}} \mathcal {F}_{x, t} \big ( \varphi _T \overline{u_{N_1}} \cdot \varphi _T \overline{v_{N_2}} \big ) \big \Vert _{\ell _n^2 L_\tau ^2 (\mathfrak {P}_N)} \\&= \bigg \Vert \langle \tau + |n|^2 \rangle ^{\frac{s}{2} - \frac{1}{3}} \widetilde{\widehat{u_{N_1}}} * \widetilde{\widehat{\varphi _T^2 v_{N_2}}} \bigg \Vert _{\ell _n^2 L_\tau ^2 (\mathfrak {P}_N)} \\&\le N^{s - \frac{2}{3}} \big \Vert \widehat{u_{N_1}} \big \Vert _{\ell _{n_1}^2 L_{\tau _1}^1} \Big \Vert \widehat{\varphi _T^2 v_{N_2}} \Big \Vert _{\ell _{n_2}^1 L_{\tau _2}^2} \\&\le _\varphi N^{s - \frac{2}{3}} N_1^{-s} \big \Vert \langle n_1 \rangle ^s \widehat{u_{N_1}} \big \Vert _{\ell _{n_1}^2 L_{\tau _1}^1} T^\varepsilon N_2 \big \Vert \langle \tau _2 + |n_2|^2 \rangle ^\varepsilon \widehat{v_{N_2}} \big \Vert _{\ell _{n_2}^2 L_{\tau _2}^2} \\&\le N^{s - \frac{2}{3}} N_1^{-s} \Vert u_{N_1} \Vert _{Z^{s, \frac{2}{3}}} T^\varepsilon N_2^{- s - \frac{1}{3} + 2\varepsilon } \Vert v_{N_2} \Vert _{Y^{s, \frac{2}{3}}} \\&\le N^{- \frac{2}{3}} N_2^{- s - \frac{1}{3} + 2\varepsilon } T^\varepsilon \Vert u_{N_1} \Vert _{Z^{s, \frac{2}{3}}} \Vert v_{N_2} \Vert _{Z^{s, \frac{2}{3}}}, \end{aligned} \end{aligned}$$

where $\varepsilon > 0$ is arbitrarily small. Since $-s - \frac{1}{3} + 2\varepsilon > 0$ given $s \le - \frac{1}{2}$, the above estimate is acceptable if $-s - 1 + 2\varepsilon < 0$, which is valid given $s > - \frac{2}{3}$ and $\varepsilon > 0$ sufficiently small.

Also, by the Cauchy-Schwarz inequality, we get

$$\begin{aligned} \big \Vert&\langle n \rangle ^s \langle \tau + |n|^2 \rangle ^{- 1} \mathcal {F}_{x, t} \big ( \varphi _T \overline{u_{N_1}} \cdot \varphi _T \overline{v_{N_2}} \big ) \big \Vert _{\ell _n^2 L_\tau ^1 (\mathfrak {P}_N)} \\&\le \big \Vert \langle n \rangle ^s \langle \tau + |n|^2 \rangle ^{- \frac{1}{3}} \mathcal {F}_{x, t} \big ( \varphi _T \overline{u_{N_1}} \cdot \varphi _T \overline{v_{N_2}} \big ) \big \Vert _{\ell _n^2 L_\tau ^2 (\mathfrak {P}_N)}, \end{aligned}$$

which can be estimated similarly as in (3.2). Combining the above two estimates, we obtain the desired inequality.

**Subcase 1.3** $$|\tau _1 + |n_1|^2| < 2^{-10} |n_1|^2$$ and $$|\tau _2 + |n_2|^2| < 2^{-10} |n_2|^2$$.

In this subcase, we need to estimate both $u_{N_1}$ and $v_{N_2}$ using the $X^{s, \frac{2}{3}}$-norm. Using the fact that $\varphi _T$ is supported on $[-1, 1]$ given $0 < T \le \frac{1}{2}$, by the Plancherel theorem, Hölder’s inequality, Lemma 2.4, and Lemma 2.3, we obtain

3.3 $$\begin{aligned} \begin{aligned} \big \Vert&\langle \tau + |n|^2 \rangle ^{\frac{s}{2} - \frac{1}{3}} \mathcal {F}_{x, t} \big ( \varphi _T \overline{u_{N_1}} \cdot \varphi _T \overline{v_{N_2}} \big ) \big \Vert _{\ell _n^2 L_\tau ^2 (\mathfrak {P}_N)} \\&\le N^{s - \frac{2}{3}} \Vert \varphi _T \overline{u_{N_1}} \cdot \varphi _T \overline{v_{N_2}} \Vert _{L^2_t ([-1, 1]; L^2_x (\mathbb {T}^2))} \\&\le N^{s - \frac{2}{3}} \Vert \varphi _T u_{N_1} \Vert _{L^4_t ([-1, 1]; L^4_x (\mathbb {T}^2))} \Vert \varphi _T v_{N_2} \Vert _{L^4_t ([-1, 1]; L^4_x (\mathbb {T}^2))} \\&\le N^{s - \frac{2}{3}} N_1^{4\varepsilon } \Vert \varphi _T u_{N_1} \Vert _{X^{0, \frac{1}{2} - \varepsilon }} N_2^{4\varepsilon } \Vert \varphi _T v_{N_2} \Vert _{X^{0, \frac{1}{2} - \varepsilon }} \\&\le _\varphi N^{s - \frac{2}{3}} N_1^{-s + 4\varepsilon } T^{\frac{\varepsilon }{2}} \Vert u_{N_1} \Vert _{X^{s, \frac{1}{2} - \frac{\varepsilon }{2}}} N_2^{-s + 4\varepsilon } T^{\frac{\varepsilon }{2}} \Vert v_{N_2} \Vert _{X^{s, \frac{1}{2} - \frac{\varepsilon }{2}}} \\&\le N^{- \frac{2}{3} + 4\varepsilon } N_2^{-s + 4\varepsilon } T^{\varepsilon } \Vert u_{N_1} \Vert _{Z^{s, \frac{2}{3}}} \Vert v_{N_2} \Vert _{Z^{s, \frac{2}{3}}}, \end{aligned} \end{aligned}$$

where $\varepsilon > 0$ is arbitrarily small. Since $s \le - \frac{1}{2} < 0$, the above estimate is acceptable if $-s - \frac{2}{3} + 8\varepsilon < 0$, which is valid given $s > - \frac{2}{3}$ and $\varepsilon > 0$ small enough.

Also, by the Cauchy-Schwarz inequality, we get

$$\begin{aligned} \big \Vert&\langle n \rangle ^s \langle \tau + |n|^2 \rangle ^{- 1} \mathcal {F}_{x, t} \big ( \varphi _T \overline{u_{N_1}} \cdot \varphi _T \overline{v_{N_2}} \big ) \big \Vert _{\ell _n^2 L_\tau ^1 (\mathfrak {P}_N)} \\&\le \big \Vert \langle n \rangle ^s \langle \tau + |n|^2 \rangle ^{- \frac{1}{3}} \mathcal {F}_{x, t} \big ( \varphi _T \overline{u_{N_1}} \cdot \varphi _T \overline{v_{N_2}} \big ) \big \Vert _{\ell _n^2 L_\tau ^2 (\mathfrak {P}_N)}, \end{aligned}$$

which can be estimated similarly as in (3.3). Combining the above two estimates, we obtain the desired inequality.

**Case 2** $$|\tau + |n|^2| < 2^{-10} |n|^2$$.

In this case, we need to evaluate the $\mathcal {F}_{x, t} \big ( \varphi _T \overline{u_{N_1}} \cdot \varphi _T \overline{v_{N_2}} \big )$ term using the $\widehat{X}^{s, \frac{2}{3}}$-norm.

We assume that $n \ne 0$. Note that if $n = 0$, we have $N = 1$ which then implies that $N_1 \le 2^5$ and $N_2 \le 2^6$, and so the estimate will follow in a similar (and much easier) manner.

We consider the following three subcases.

**Subcase 2.1** $$|\tau _1 + |n_1|^2| \ge 2^{-10} |n_1|^2$$ and $$|\tau _2 + |n_2|^2| \ge 2^{-10} |n_2|^2$$.

In this subcase, we need to estimate both $u_{N_1}$ and $v_{N_2}$ using the $Y^{s, \frac{2}{3}}$-norm. By Hölder’s inequality, Young’s convolution inequality, and Lemma 2.3, we have

$$\begin{aligned} \big \Vert&\langle n \rangle ^s \langle \tau + |n|^2 \rangle ^{- \frac{1}{3}} \mathcal {F}_{x, t} \big ( \varphi _T \overline{u_{N_1}} \cdot \varphi _T \overline{v_{N_2}} \big ) \big \Vert _{\ell _n^2 L_\tau ^2 (\mathfrak {P}_N)} \\&= \bigg \Vert \langle n \rangle ^s \langle \tau + |n|^2 \rangle ^{- \frac{1}{3}} \widetilde{\widehat{u_{N_1}}} * \widetilde{\widehat{\varphi _T^2 v_{N_2}}} \bigg \Vert _{\ell _n^2 L_\tau ^2 (\mathfrak {P}_N)} \\&\le \big \Vert \langle n \rangle ^s \langle \tau + |n|^2 \rangle ^{- \frac{1}{3}} \big \Vert _{\ell _n^2 L_\tau ^2 (\mathfrak {P}_N)} \big \Vert \widehat{u_{N_1}} \big \Vert _{\ell _{n_1}^2 L_{\tau _1}^2} \Big \Vert \widehat{\varphi _T^2 v_{N_2}} \Big \Vert _{\ell _{n_2}^2 L_{\tau _2}^2} \\&\le _\varphi N^{s + 1} N^{\frac{1}{3}} N_1^{- s - \frac{4}{3}} \Vert u_{N_1} \Vert _{Y^{s, \frac{2}{3}}} T^\varepsilon \big \Vert \langle \tau _2 + |n_2|^2 \rangle ^\varepsilon \widehat{v_{N_2}} \big \Vert _{\ell _{n_2}^2 L_{\tau _2}^2} \\&\le T^{\varepsilon } \Vert u_{N_1} \Vert _{Z^{s, \frac{2}{3}}} N_2^{-s - \frac{4}{3} + 2 \varepsilon } \Vert v_{N_2} \Vert _{Y^{s, \frac{2}{3}}} \\&\le N_2^{-s - \frac{4}{3} + 2\varepsilon } T^{\varepsilon } \Vert u_{N_1} \Vert _{Z^{s, \frac{2}{3}}} \Vert v_{N_2} \Vert _{Z^{s, \frac{2}{3}}}, \end{aligned}$$

where $\varepsilon > 0$ is arbitrarily small. The above estimate is acceptable if $-s - \frac{4}{3} + 2\varepsilon < 0$, which is valid given $s > - \frac{2}{3}$ and $\varepsilon > 0$ sufficiently small.

**Subcase 2.2** $$| \tau _1 + |n_1|^2 | \ge 2^{-10} |n_1|^2$$ and $$| \tau _2 + |n_2|^2 | < 2^{-10} |n_2|^2$$.

In this subcase, we need to estimate $u_{N_1}$ using the $Y^{s, \frac{2}{3}}$-norm and estimate $v_{N_2}$ using the $X^{s, \frac{2}{3}}$-norm. By duality and the Cauchy-Schwarz inequality, we have

3.4 $$\begin{aligned} \begin{aligned} \big \Vert&\langle n \rangle ^s \langle \tau + |n|^2 \rangle ^{- \frac{1}{3}} \mathcal {F}_{x, t} \big ( \varphi _T \overline{u_{N_1}} \cdot \varphi _T \overline{v_{N_2}} \big ) \big \Vert _{\ell _n^2 L_\tau ^2 (\mathfrak {P}_N)} \\&\le N^s \sup _{\Vert h \Vert _{\ell _n^2 L_\tau ^2 (\mathfrak {P}_N)} \le 1} \bigg | \sum _{\begin{array}{c} n, n_1, n_2 \in \mathbb {Z}^2 \\ n + n_1 + n_2 = 0 \end{array}} \iint _{\tau + \tau _1 + \tau _2 = 0} \widehat{\varphi _T u_{N_1}} (n_1, \tau _1) \widehat{\varphi _T v_{N_2}} (n_2, \tau _2) \\&\qquad \times \frac{h(n, \tau )}{\langle \tau + |n|^2 \rangle ^{\frac{1}{3}}} d\tau d\tau _1 \bigg | \\&\le N^s \big \Vert \widehat{\varphi _T u_{N_1}} \big \Vert _{\ell _{n_1}^2 L_{\tau _1}^2} \sup _{\Vert h \Vert _{\ell _n^2 L_\tau ^2 (\mathfrak {P}_N)} \le 1} \bigg \Vert \sum _{\begin{array}{c} n, n_2 \in \mathbb {Z}^2 \\ n + n_1 + n_2 = 0 \end{array}} \int _{\tau + \tau _1 + \tau _2 = 0} \widehat{\varphi _T v_{N_2}} (n_2, \tau _2) \\&\qquad \times \frac{h(n, \tau )}{\langle \tau + |n|^2 \rangle ^{\frac{1}{3}}} d\tau \bigg \Vert _{\ell _{n_1}^2 L_{\tau _1}^2}. \end{aligned} \end{aligned}$$

Let $w_N$ be a space-time distribution that satisfy $\widehat{w_N} (n, \tau ) = h(n, \tau ) / \langle \tau + |n|^2 \rangle ^{\frac{1}{3}}$. Then, using the fact that $\varphi _T$ is supported on $[-1, 1]$ given $0 < T \le \frac{1}{2}$, by the Plancherel theorem, Hölder’s inequality, Lemma 2.4, and Lemma 2.3, we have

$$\begin{aligned} \bigg \Vert&\sum _{\begin{array}{c} n, n_2 \in \mathbb {Z}^2 \\ n + n_1 + n_2 = 0 \end{array}} \int _{\tau + \tau _1 + \tau _2 = 0} \widehat{\varphi _T v_{N_2}} (n_2, \tau _2) \frac{h(n, \tau )}{\langle \tau + |n|^2 \rangle ^{\frac{1}{3}}} d\tau \bigg \Vert _{\ell _{n_1}^2 L_{\tau _1}^2} \\&= \Vert \varphi _T v_{N_2} \widetilde{w_N} \Vert _{L^2_t ([-1, 1]; L^2_x (\mathbb {T}^2))} \\&\le \Vert \varphi _T v_{N_2} \Vert _{L^4_t ([-1, 1]; L^4_x (\mathbb {T}^2))} \Vert w_N \Vert _{L^4_t ([-1, 1]; L^4_x (\mathbb {T}^2))} \\&\le N_2^{4 \varepsilon } \Vert \varphi _T v_{N_2} \Vert _{X^{0, \frac{1}{2} - \varepsilon }} N^{\frac{1}{3} + \varepsilon } \Vert w_N \Vert _{X^{0, \frac{1}{3}}} \\&\le _\varphi N_2^{- s + 4\varepsilon } T^{\frac{\varepsilon }{2}} \Vert v_{N_2} \Vert _{X^{s, \frac{2}{3}}} N^{\frac{1}{3} + \varepsilon } \Vert h \Vert _{\ell _n^2 L_\tau ^2 (\mathfrak {P}_N)}, \end{aligned}$$

where $\varepsilon > 0$ is arbitrarily small. Thus, continuing with (3.4), we use Lemma 2.3 to obtain

$$\begin{aligned} \big \Vert&\langle n \rangle ^s \langle \tau + |n|^2 \rangle ^{- \frac{1}{3}} \mathcal {F}_{x, t} \big ( \varphi _T \overline{u_{N_1}} \cdot \varphi _T \overline{v_{N_2}} \big ) \big \Vert _{\ell _n^2 L_\tau ^2 (\mathfrak {P}_N)} \\&\le _\varphi N^{s + \frac{1}{3} + \varepsilon } \big \Vert \widehat{\varphi _T u_{N_1}} \big \Vert _{\ell _{n_1}^2 L_{\tau _1}^2} N_2^{-s + 4\varepsilon } T^{\frac{\varepsilon }{2}} \Vert v_{N_2} \Vert _{X^{s, \frac{2}{3}}} \\&\le _\varphi N^{s + \frac{1}{3} + \varepsilon } N_2^{-s + 4\varepsilon } T^{\varepsilon } \big \Vert \langle \tau _1 + |n_1|^2 \rangle ^\frac{\varepsilon }{2} \widehat{u_{N_1}} \big \Vert _{\ell _{n_1}^2 L_{\tau _1}^2} \Vert v_{N_2} \Vert _{Z^{s, \frac{2}{3}}} \\&\le N^{s + \frac{1}{3} + \varepsilon } N_2^{-s + 4\varepsilon } T^{\varepsilon } N_1^{-s - \frac{4}{3} + \varepsilon } \Vert u_{N_1} \Vert _{Y^{s, \frac{2}{3}}} \Vert v_{N_2} \Vert _{Z^{s, \frac{2}{3}}} \\&\le N^{- 1 + 2 \varepsilon } N_2^{-s + 4\varepsilon } T^{\varepsilon } \Vert u_{N_1} \Vert _{Z^{s, \frac{2}{3}}} \Vert v_{N_2} \Vert _{Z^{s, \frac{2}{3}}}. \end{aligned}$$

Since $s < 0$, the above estimate is acceptable if $-s - 1 + 6 \varepsilon < 0$, which is valid given $s > - \frac{2}{3}$ and $\varepsilon > 0$ small enough.

**Subcase 2.3** $$|\tau _1 + |n_1|^2| < 2^{-10} |n_1|^2$$.

In this subcase, we first note that

$$\begin{aligned} \tau< 2^{-10} |n|^2 - |n|^2 \quad \text {and} \quad \tau _1 < 2^{-10} |n_1|^2 - |n_1|^2. \end{aligned}$$

Note that since we assumed $n \ne 0$, we have

$$\begin{aligned} \tau _2 = - \tau - \tau _1> |n|^2 - 2^{-10} |n|^2 + |n_1|^2 - 2^{-10} |n_1|^2 > \frac{1}{2} |n|^2. \end{aligned}$$

Thus, we have

3.5 $$\begin{aligned} |\tau _2 + |n_2|^2| \ge N^2 \end{aligned}$$

and $$|\tau _2 + |n_2|^2| \ge |n_2|^2 > 2^{-10} |n_2|^2$$.

We need to estimate $u_{N_1}$ using the $X^{s, \frac{2}{3}}$-norm and estimate $v_{N_2}$ using the $Y^{s, \frac{2}{3}}$-norm. By using similar steps as in Subcase 2.2 by switching the roles of $u_{N_1}$ and $v_{N_2}$ along with the additional condition (3.5), we obtain

$$\begin{aligned} \big \Vert&\langle n \rangle ^s \langle \tau + |n|^2 \rangle ^{- \frac{1}{3}} \mathcal {F}_{x, t} \big ( \varphi _T \overline{u_{N_1}} \cdot \varphi _T \overline{v_{N_2}} \big ) \big \Vert _{\ell _n^2 L_\tau ^2 (\mathfrak {P}_N)} \\&\le _\varphi N^{s + \frac{1}{3} + \varepsilon } N_1^{-s + 4\varepsilon } T^{\frac{\varepsilon }{2}} \Vert u_{N_1} \Vert _{X^{s, \frac{2}{3}}} \big \Vert \widehat{\varphi _T v_{N_2}} \big \Vert _{\ell _{n_2}^2 L_{\tau _2}^2} \\&\le _\varphi N^{\frac{1}{3} + 5 \varepsilon } T^\varepsilon \Vert u_{N_1} \Vert _{Z^{s, \frac{2}{3}}} \big \Vert \langle \tau _2 + |n_2|^2 \rangle ^{\frac{\varepsilon }{2}} \widehat{v_{N_2}} \big \Vert _{\ell _{n_2}^2 L_{\tau _2}^2} \\&\le N^{\frac{1}{3} + 5 \varepsilon } T^\varepsilon \Vert u_{N_1} \Vert _{Z^{s, \frac{2}{3}}} N^{-s - \frac{4}{3} + \varepsilon } \Vert v_{N_2} \Vert _{Y^{s, \frac{2}{3}}} \\&\le N^{-s - 1 + 6 \varepsilon } T^\varepsilon \Vert u_{N_1} \Vert _{Z^{s, \frac{2}{3}}} \Vert v_{N_2} \Vert _{Z^{s, \frac{2}{3}}}. \end{aligned}$$

where $\varepsilon > 0$ is arbitrarily small. The above estimate is acceptable if $-s - 1 + 6 \varepsilon < 0$, which is valid given $s > - \frac{2}{3}$ and $\varepsilon > 0$ small enough.

Thus, we have finished our proof. $\square$

We now show the following “high-high interaction” estimate.

### Lemma 3.3

Let $-\frac{2}{3} < s \le -\frac{1}{2}$ and $0 < T \le \frac{1}{2}$. Let *N*, $N_1$, and $N_2$ be dyadic numbers such that $\frac{1}{2} N_1 \le N_2 \le 2 N_1$ and $N < 2^{-5} N_1$. Let $\varphi : \mathbb {R}\rightarrow [0, 1]$ be a smooth function such that $\varphi \equiv 1$ on $[-1, 1]$ and $\varphi \equiv 0$ outside of $[-2, 2]$, and let $\varphi _T (t):= \varphi (t / T)$. Then, we have

$$\begin{aligned} \big \Vert \langle \tau + |n|^2 \rangle ^{-1} \mathcal {F}_{x, t} \big ( \varphi _T \overline{u_{N_1}} \cdot \varphi _T \overline{v_{N_2}} \big ) (n, \tau ) \big \Vert _{\widehat{Z}^{s, \frac{2}{3}} (\mathfrak {P}_N)} \le _\varphi N^{-\delta } T^\theta \Vert u_{N_1} \Vert _{Z^{s, \frac{2}{3}}} \Vert v_{N_2} \Vert _{Z^{s, \frac{2}{3}}} \end{aligned}$$

for some $\delta > 0$ and $\theta > 0$.

### Proof

As in the proof of the previous lemma, we use $(n_1, \tau _1)$ as the variables of $\overline{\varphi _T u_{N_1}}$ or $\overline{u_{N_1}}$, and $(n_2, \tau _2)$ as the variables of $\overline{\varphi _T v_{N_2}}$ or $\overline{v_{N_2}}$. Note that we have the relations $\tau + \tau _1 + \tau _2 = 0$ and $n + n_1 + n_2 = 0$. Also, the assumptions on the sizes of *N*, $N_1$, and $N_2$ ensure that $n_1 \ne 0$ and $n_2 \ne 0$. We also recall the notation $\widetilde{f} (x) = f(-x)$.

We consider the following four main cases.

**Case 1** $$|\tau + |n|^2| \ge 2^{-10} |n_1|^2$$.

In this case, we have $|\tau + |n|^2| \ge 2^{-10} |n_1|^2 \ge 2^{-10} |n|^2$ given $N < 2^{-5} N_1$, so that we need to evaluate the $\mathcal {F}_{x, t} \big ( \varphi _T \overline{u_{N_1}} \cdot \varphi _T \overline{v_{N_2}} \big )$ term using the $\widehat{Y}^{s, \frac{2}{3}}$-norm, and we need to evaluate both the $\ell _n^2 L_\tau ^1$ term and the $\ell _n^2 L_\tau ^2$ term. We consider the following three subcases.

**Subcase 1.1** $$|\tau _1 + |n_1|^2| \ge 2^{-10} |n_1|^2$$.

In this subcase, we need to estimate $u_{N_1}$ using the $Y^{s, \frac{2}{3}}$-norm. By Young’s convolution inequality, Lemma 2.3, the Cauchy-Schwarz inequality, and Lemma 2.5, we obtain

$$\begin{aligned} \big \Vert&\langle \tau + |n|^2 \rangle ^{\frac{s}{2} - \frac{1}{3}} \mathcal {F}_{x, t} \big ( \varphi _T \overline{u_{N_1}} \cdot \varphi _T \overline{v_{N_2}} \big ) \big \Vert _{\ell _n^2 L_\tau ^2 (\mathfrak {P}_N)} \\&= \bigg \Vert \langle \tau + |n|^2 \rangle ^{\frac{s}{2} - \frac{1}{3}} \widetilde{\widehat{\varphi _T^2 u_{N_1}}} * \widetilde{\widehat{v_{N_2}}} \bigg \Vert _{\ell _n^2 L_\tau ^2 (\mathfrak {P}_N)} \\&\le N_1^{s - \frac{2}{3}} \Big \Vert \widehat{\varphi _T^2 u_{N_1}} \Big \Vert _{\ell _{n_1}^2 L_{\tau _1}^2} \big \Vert \widehat{v_{N_2}} \big \Vert _{\ell _{n_2}^1 L_{\tau _2}^1} \\&\le _\varphi N_1^{s - \frac{2}{3}} T^\varepsilon \big \Vert \langle \tau _1 + |n_1|^2 \rangle ^\varepsilon \widehat{u_{N_1}} \big \Vert _{\ell _{n_1}^2 L_{\tau _1}^2} N_2^{-s + 1} \big \Vert \langle n_2 \rangle ^s \widehat{v_{N_2}} \big \Vert _{\ell _{n_2}^2 L_{\tau _2}^1} \\&\le N_1^{s - \frac{2}{3}} T^\varepsilon N_1^{-s - \frac{4}{3} + 2\varepsilon } \Vert u_{N_1} \Vert _{Y^{s, \frac{2}{3}}} N_2^{-s + 1} \Vert v_{N_2} \Vert _{Z^{s, \frac{2}{3}}} \\&\le N_1^{-s - 1 + 2 \varepsilon } T^\varepsilon \Vert u_{N_1} \Vert _{Z^{s, \frac{2}{3}}} \Vert v_{N_2} \Vert _{Z^{s, \frac{2}{3}}}, \end{aligned}$$

which is acceptable given $s > - \frac{2}{3}$ and $\varepsilon > 0$ sufficiently small.

Also, by Hölder’s inequality, Young’s convolution inequality, Lemma 2.3, and Lemma 2.5, we have

$$\begin{aligned} \big \Vert&\langle n \rangle ^s \langle \tau + |n|^2 \rangle ^{-1} \mathcal {F}_{x, t} \big ( \varphi _T \overline{u_{N_1}} \cdot \varphi _T \overline{v_{N_2}} \big ) \big \Vert _{\ell _n^2 L_\tau ^1 (\mathfrak {P}_N)} \\&= \bigg \Vert \langle n \rangle ^s \langle \tau + |n|^2 \rangle ^{-1} \widetilde{\widehat{\varphi _T^2 u_{N_1}}} * \widetilde{\widehat{v_{N_2}}} \bigg \Vert _{\ell _n^2 L_\tau ^1 (\mathfrak {P}_N)} \\&\le N_1^{- 1 + 2 \varepsilon } \big \Vert \langle n \rangle ^s \langle \tau + |n|^2 \rangle ^{-\frac{1}{2} - \varepsilon } \big \Vert _{\ell _n^2 L_\tau ^2 (\mathfrak {P}_N)} \Big \Vert \widehat{\varphi _T^2 u_{N_1}} \Big \Vert _{\ell _{n_1}^2 L_{\tau _1}^2} \big \Vert \widehat{v_{N_2}} \big \Vert _{\ell _{n_2}^2 L_{\tau _2}^1} \\&\le _\varphi N_1^{- 1 + 2 \varepsilon } N^{s + 1} T^\varepsilon \big \Vert \langle \tau _1 + |n_1|^2 \rangle ^\varepsilon \widehat{u_{N_1}} \big \Vert _{\ell _{n_2}^2 L_{\tau _2}^2} N_2^{-s} \big \Vert \langle n_2 \rangle ^s \widehat{v_{N_2}} \big \Vert _{\ell _{n_2}^2 L_{\tau _2}^1} \\&\le N^{s + 1} N_1^{-s - 1 + 2\varepsilon } T^\varepsilon N_1^{-s - \frac{4}{3} + 2 \varepsilon } \Vert u_{N_1} \Vert _{Y^{s, \frac{2}{3}}} \Vert v_{N_2} \Vert _{Z^{s, \frac{2}{3}}} \\&\le N^{s + 1} N_1^{-2s - \frac{7}{3} + 4 \varepsilon } T^\varepsilon \Vert u_{N_1} \Vert _{Z^{s, \frac{2}{3}}} \Vert v_{N_2} \Vert _{Z^{s, \frac{2}{3}}}, \end{aligned}$$

where $\varepsilon > 0$ is arbitrarily small. Since $s + 1 > 0$ given $s > - \frac{2}{3}$, the above estimate is acceptable if $-s - \frac{4}{3} + 4 \varepsilon < 0$, which is valid given $s > -\frac{2}{3}$ and $\varepsilon > 0$ small enough. Combining the above two estimates, we obtain the desired inequality.

**Subcase 1.2** $$|\tau _2 + |n_2|^2| \ge 2^{-10} |n_2|^2$$.

This subcase is similar to Subcase 1.1 by switching the roles of $u_{N_1}$ and $v_{N_2}$, and so we omit details.

**Subcase 1.3** $$|\tau _1 + |n_1|^2| < 2^{-10} |n_1|^2$$ and $$|\tau _2 + |n_2|^2| < 2^{-10} |n_2|^2$$.

In this subcase, we need to estimate both $u_{N_1}$ and $v_{N_2}$ using the $X^{s, \frac{2}{3}}$-norm. Using the fact that $\varphi _T$ is supported on $[-1, 1]$ given $0 < T \le \frac{1}{2}$, by the Plancherel theorem, Hölder’s inequality, Lemma 2.4, and Lemma 2.3, we obtain

$$\begin{aligned} \big \Vert&\langle \tau + |n|^2 \rangle ^{\frac{s}{2} - \frac{1}{3}} \mathcal {F}_{x, t} \big ( \varphi _T \overline{u_{N_1}} \cdot \varphi _T \overline{v_{N_2}} \big ) \big \Vert _{\ell _n^2 L_\tau ^2 (\mathfrak {P}_N)} \\&\le N_1^{s - \frac{2}{3}} \Vert \varphi _T \overline{u_{N_1}} \cdot \varphi _T \overline{v_{N_2}} \Vert _{L^2_t ([-1, 1]; L^2_x (\mathbb {T}^2))} \\&\le N_1^{s - \frac{2}{3}} \Vert \varphi _T u_{N_1} \Vert _{L^4_t ([-1, 1]; L^4_x (\mathbb {T}^2))} \Vert \varphi _T v_{N_2} \Vert _{L^4_t ([-1, 1]; L^4_x (\mathbb {T}^2))} \\&\le N_1^{s - \frac{2}{3}} N_1^{4 \varepsilon } \Vert \varphi _T u_{N_1} \Vert _{X^{0, \frac{1}{2} - \varepsilon }} N_2^{4 \varepsilon } \Vert \varphi _T v_{N_2} \Vert _{X^{0, \frac{1}{2} - \varepsilon }} \\&\le _\varphi N_1^{s - \frac{2}{3}} N_1^{-s + 4 \varepsilon } T^{\frac{\varepsilon }{2}} \Vert u_{N_1} \Vert _{X^{s, \frac{2}{3}}} N_2^{-s + 4 \varepsilon } T^{\frac{\varepsilon }{2}} \Vert v_{N_2} \Vert _{X^{s, \frac{2}{3}}} \\&\le N_1^{-s - \frac{2}{3} + 8 \varepsilon } T^\varepsilon \Vert u_{N_1} \Vert _{Z^{s, \frac{2}{3}}} \Vert v_{N_2} \Vert _{Z^{s, \frac{2}{3}}}, \end{aligned}$$

where $\varepsilon > 0$ is arbitrarily small. The above estimate is acceptable if $-s - \frac{2}{3} + 8 \varepsilon < 0$, which is valid given $s > -\frac{2}{3}$ and $\varepsilon > 0$ small enough.

Regarding the $\ell _n^2 L_\tau ^1$ norm of the $\mathcal {F}_{x, t} \big ( \varphi _T \overline{u_{N_1}} \cdot \varphi _T \overline{v_{N_2}} \big )$ term, we first let $\varepsilon _1, \varepsilon _2 > 0$ satisfying

$$\begin{aligned} 1 + \frac{1}{1 + \varepsilon _1} = \frac{2}{1 + \varepsilon _2}. \end{aligned}$$

Note that both $\varepsilon _1$ and $\varepsilon _2$ can be arbitrarily small. By Hölder’s inequality, Young’s convolution inequality, Hölder’s inequalities twice, and Lemma 2.3, we have

$$\begin{aligned} \big \Vert&\langle n \rangle ^s \langle \tau + |n|^2 \rangle ^{- 1} \mathcal {F}_{x, t} \big ( \varphi _T \overline{u_{N_1}} \cdot \varphi _T \overline{v_{N_2}} \big ) \big \Vert _{\ell _n^2 L_\tau ^1 (\mathfrak {P}_N)} \\&= \Big \Vert \langle n \rangle ^s \langle \tau + |n|^2 \rangle ^{- 1} \widetilde{\widehat{\varphi _T u_{N_1}}} * \widetilde{\widehat{\varphi _T v_{N_2}}} \Big \Vert _{\ell _n^2 L_\tau ^1 (\mathfrak {P}_N)} \\&\le N_1^{- 2 + 2 \varepsilon _1} \big \Vert \langle n \rangle ^s \langle \tau + |n|^2 \rangle ^{- \varepsilon _1} \big \Vert _{\ell _n^2 L_\tau ^{(1 + \varepsilon _1) / \varepsilon _1} (\mathfrak {P}_N)} \big \Vert \widehat{\varphi _T u_{N_1}} \big \Vert _{\ell _{n_1}^2 L_{\tau _1}^{1 + \varepsilon _2}} \big \Vert \widehat{\varphi _T v_{N_2}} \big \Vert _{\ell _{n_2}^2 L_{\tau _2}^{1 + \varepsilon _2}} \\&\le N_1^{- 2 + 2 \varepsilon _1} N^{s + 1} \big \Vert \langle \tau _1 + |n_1|^2 \rangle ^{\frac{1}{2 + 2 \varepsilon _1} +} \widehat{\varphi _T u_{N_1}} \big \Vert _{\ell _{n_1}^2 L_{\tau _1}^2} \big \Vert \langle \tau _2 + |n_2|^2 \rangle ^{\frac{1}{2 + 2 \varepsilon _1} +} \widehat{\varphi _T v_{N_2}} \big \Vert _{\ell _{n_2}^2 L_{\tau _2}^2} \\&\le _\varphi N^{s + 1} N_1^{- 2 + 2 \varepsilon _1} T^\theta N_1^{-s} \Vert u_{N_1} \Vert _{X^{s, \frac{2}{3}}} T^{\theta } N_2^{-s} \Vert v_{N_2} \Vert _{X^{s, \frac{2}{3}}} \\&\le N^{s + 1} N_1^{- 2s - 2 + 2 \varepsilon _1} T^{2 \theta } \Vert u_{N_1} \Vert _{Z^{s, \frac{2}{3}}} \Vert v_{N_2} \Vert _{Z^{s, \frac{2}{3}}} \end{aligned}$$

for some $\theta > 0$. Since $s + 1 > 0$ given $s > - \frac{2}{3}$, the above estimate is acceptable if $-s - 1 + 2 \varepsilon _1 < 0$, which is valid given $s > - \frac{2}{3}$ and $\varepsilon _1 > 0$ close enough to 0. Combining the above two estimates, we obtain the desired inequality.

**Case 2** $$|\tau + |n|^2| < 2^{-10} |n_1|^2$$, $$|\tau _1 + |n_1|^2| \ge 2^{-10} |n_1|^2$$, and $$|\tau _2 + |n_2|^2| \ge 2^{-10} |n_2|^2$$.

In this case, we need to estimate both $u_{N_1}$ and $v_{N_2}$ using the $Y^{s, \frac{2}{3}}$-norm. We consider the following two subcases.

**Subcase 2.1** $$|\tau + |n|^2| < 2^{-10} |n|^2$$.

In this subcase, we need to evaluate the $\mathcal {F}_{x, t} \big ( \varphi _T \overline{u_{N_1}} \cdot \varphi _T \overline{v_{N_2}} \big )$ term using the $\widehat{X}^{s, \frac{2}{3}}$-norm. By Hölder’s inequality, Young’s convolution inequality, and Lemma 2.3, we have

$$\begin{aligned} \big \Vert&\langle n \rangle ^s \langle \tau + |n|^2 \rangle ^{- \frac{1}{3}} \mathcal {F}_{x, t} \big ( \varphi _T \overline{u_{N_1}} \cdot \varphi _T \overline{v_{N_2}} \big ) \big \Vert _{\ell _n^2 L_\tau ^2 (\mathfrak {P}_N)} \\&= \Big \Vert \langle n \rangle ^s \langle \tau + |n|^2 \rangle ^{- \frac{1}{3}} \widetilde{\widehat{\varphi _T u_{N_1}}} * \widetilde{\widehat{\varphi _T v_{N_2}}} \Big \Vert _{\ell _n^2 L_\tau ^2 (\mathfrak {P}_N)} \\&\le \big \Vert \langle n \rangle ^s \langle \tau + |n|^2 \rangle ^{- \frac{1}{3}} \big \Vert _{\ell _n^2 L_\tau ^2 (\mathfrak {P}_N)} \big \Vert \widehat{\varphi _T u_{N_1}} \big \Vert _{\ell _{n_1}^2 L_{\tau _1}^2} \big \Vert \widehat{\varphi _T v_{N_2}} \big \Vert _{\ell _{n_2}^2 L_{\tau _2}^2} \\&\le _\varphi N^{s + 1} N^{\frac{1}{3}} T^\varepsilon \big \Vert \langle \tau _1 + |n_1|^2 \rangle ^\varepsilon \widehat{u_{N_1}} \big \Vert _{\ell _{n_1}^2 L_{\tau _1}^2} T^\varepsilon \big \Vert \langle \tau _2 + |n_2|^2 \rangle ^\varepsilon \widehat{v_{N_2}} \big \Vert _{\ell _{n_2}^2 L_{\tau _2}^2} \\&\le N^{s + \frac{4}{3}} T^\varepsilon N_1^{-s - \frac{4}{3} + 2 \varepsilon } \Vert u_{N_1} \Vert _{Y^{s, \frac{2}{3}}} T^\varepsilon N_2^{-s - \frac{4}{3} + 2 \varepsilon } \Vert v_{N_2} \Vert _{Y^{s, \frac{2}{3}}} \\&\le N^{s + \frac{4}{3}} N_1^{-2s - \frac{8}{3} + 4 \varepsilon } T^{2 \varepsilon } \Vert u_{N_1} \Vert _{Z^{s, \frac{2}{3}}} \Vert v_{N_2} \Vert _{Z^{s, \frac{2}{3}}}, \end{aligned}$$

where $\varepsilon > 0$ is arbitrarily small. Since $s > -\frac{2}{3}$, we have $s + \frac{4}{3} > 0$. Thus, the above estimate is acceptable if $- s - \frac{4}{3} + 4 \varepsilon < 0$, which is valid given $s > -\frac{2}{3}$.

**Subcase 2.2** $$2^{-10} |n|^2 \le |\tau + |n|^2| < 2^{-10} |n_1|^2$$.

In this subcase, we need to evaluate the $\mathcal {F}_{x, t} \big ( \varphi _T \overline{u_{N_1}} \cdot \varphi _T \overline{v_{N_2}} \big )$ term using the $\widehat{Y}^{s, \frac{2}{3}}$-norm. By Hölder’s inequality, Young’s convolution inequality, and Lemma 2.3, we have

$$\begin{aligned} \begin{aligned} \big \Vert&\langle \tau + |n|^2 \rangle ^{\frac{s}{2} - \frac{1}{3}} \mathcal {F}_{x, t} \big ( \varphi _T \overline{u_{N_1}} \cdot \varphi _T \overline{v_{N_2}} \big ) \big \Vert _{\ell _n^2 L_\tau ^2 (\mathfrak {P}_N)} \\&= \Big \Vert \langle \tau + |n|^2 \rangle ^{\frac{s}{2} - \frac{1}{3}} \widetilde{\widehat{\varphi _T u_{N_1}}} * \widetilde{\widehat{\varphi _T v_{N_2}}} \Big \Vert _{\ell _n^2 L_\tau ^2 (\mathfrak {P}_N)} \\&\le \big \Vert \langle \tau + |n|^2 \rangle ^{\frac{s}{2} - \frac{1}{3}} \big \Vert _{\ell _n^2 L_\tau ^2 (\mathfrak {P}_N)} \big \Vert \widehat{\varphi _T u_{N_1}} \big \Vert _{\ell _{n_1}^2 L_{\tau _1}^2} \big \Vert \widehat{ \varphi _T v_{N_2} } \big \Vert _{\ell _{n_2}^2 L_{\tau _2}^2} \\&\le _\varphi N^{s + \frac{1}{3} + 2 \varepsilon } \big \Vert \langle \tau + |n|^2 \rangle ^{-\frac{1}{2} - \varepsilon } \big \Vert _{\ell _n^2 L_\tau ^2 (\mathfrak {P}_N)} \\&\qquad \times T^\varepsilon \big \Vert \langle \tau _1 + |n_1|^2 \rangle ^\varepsilon \widehat{u_{N_1}} \big \Vert _{\ell _{n_1}^2 L_{\tau _1}^2} T^\varepsilon \big \Vert \langle \tau _2 + |n_2|^2 \rangle ^\varepsilon \widehat{v_{N_2}} \big \Vert _{\ell _{n_2}^2 L_{\tau _2}^2} \\&\le N^{s + \frac{4}{3} + 2 \varepsilon } T^{2 \varepsilon } N_1^{-s - \frac{4}{3} + 2\varepsilon } \Vert u_{N_1} \Vert _{Y^{s, \frac{2}{3}}} N_2^{-s - \frac{4}{3} + 2\varepsilon } \Vert v_{N_2} \Vert _{Y^{s, \frac{2}{3}}} \\&\le N^{s + \frac{4}{3} + 2 \varepsilon } N_1^{-2s - \frac{8}{3} + 4 \varepsilon } T^{2 \varepsilon } \Vert u_{N_1} \Vert _{Z^{s, \frac{2}{3}}} \Vert v_{N_2} \Vert _{Z^{s, \frac{2}{3}}}, \end{aligned} \end{aligned}$$

where $\varepsilon > 0$ is arbitrarily small. Note that the second inequality is valid since $s + \frac{1}{3} + 2 \varepsilon < 0$ given $s \le - \frac{1}{2}$ and $\varepsilon > 0$ small enough. Since $s + \frac{4}{3} + 2 \varepsilon > 0$ given $s > -\frac{2}{3}$, the above estimate is acceptable if $-s - \frac{4}{3} + 6 \varepsilon < 0$, which is valid given $s > -\frac{2}{3}$.

Also, by the Cauchy-Schwarz inequality, Hölder’s inequality, Young’s convolution inequality, and Lemma 2.3, we get

$$\begin{aligned} \big \Vert&\langle n \rangle ^s \langle \tau + |n|^2 \rangle ^{- 1} \mathcal {F}_{x, t} \big ( \varphi _T \overline{u_{N_1}} \cdot \varphi _T \overline{v_{N_2}} \big ) \big \Vert _{\ell _n^2 L_\tau ^1 (\mathfrak {P}_N)} \\&\le \Big \Vert \langle n \rangle ^s \langle \tau + |n|^2 \rangle ^{- \frac{1}{2} + \varepsilon } \widetilde{\widehat{\varphi _T u_{N_1}}} * \widetilde{\widehat{\varphi _T v_{N_2}}} \Big \Vert _{\ell _n^2 L_\tau ^2 (\mathfrak {P}_N)} \\&\le N^s N_1^{4 \varepsilon } \big \Vert \langle \tau + |n|^2 \rangle ^{-\frac{1}{2} - \varepsilon } \big \Vert _{\ell _n^2 L_\tau ^2 (\mathfrak {P}_N)} \big \Vert \widehat{\varphi _T u_{N_1}} \big \Vert _{\ell _{n_1}^2 L_{\tau _1}^2} \big \Vert \widehat{ \varphi _T v_{N_2} } \big \Vert _{\ell _{n_2}^2 L_{\tau _2}^2} \\&\le _\varphi N^{s + 1} N_1^{4 \varepsilon } T^\varepsilon \big \Vert \langle \tau _1 + |n_1|^2 \rangle ^\varepsilon \widehat{u_{N_1}} \big \Vert _{\ell _{n_1}^2 L_{\tau _1}^2} T^\varepsilon \big \Vert \langle \tau _2 + |n_2|^2 \rangle ^\varepsilon \widehat{v_{N_2}} \big \Vert _{\ell _{n_2}^2 L_{\tau _2}^2} \\&\le N^{s + 1} N_1^{4 \varepsilon } T^{2 \varepsilon } N_1^{-s - \frac{4}{3} + 2 \varepsilon } \Vert u_{N_1} \Vert _{Y^{s, \frac{2}{3}}} N_2^{-s - \frac{4}{3} + 2\varepsilon } \Vert v_{N_2} \Vert _{Y^{s, \frac{2}{3}}} \\&\le N^{s + 1} N_1^{-2s - \frac{8}{3} + 8 \varepsilon } T^{2 \varepsilon } \Vert u_{N_1} \Vert _{Z^{s, \frac{2}{3}}} \Vert v_{N_2} \Vert _{Z^{s, \frac{2}{3}}}, \end{aligned}$$

where $\varepsilon > 0$ is arbitrarily small. Since $s + 1 > 0$ given $s > -\frac{2}{3}$, the above estimate is acceptable if $-s - \frac{5}{3} + 8 \varepsilon < 0$, which is valid given $s > -\frac{2}{3}$ and $\varepsilon > 0$ small enough. Combining the above two estimates, we obtain the desired inequality.

**Case 3** $$|\tau + |n|^2| < 2^{-10} |n_1|^2$$ and $$|\tau _1 + |n_1|^2| < 2^{-10} |n_1|^2$$.

In this case, we need to estimate $u_{N_1}$ using the $X^{s, \frac{2}{3}}$-norm. Note that we have

$$\begin{aligned} \tau _2&= - \tau - \tau _1 \\&= (-\tau - |n|^2) + (-\tau _1 - |n_1|^2) + |n|^2 + |n_1|^2 \\&> - 2^{-10} |n_1|^2 - 2^{-10} |n_1|^2 + |n_1|^2 \\&> 0, \end{aligned}$$

and so $|\tau _2 + |n_2|^2|> |n_2|^2 > 2^{-10} |n_2|^2$. Thus, we need to estimate $v_{N_2}$ using the $Y^{s, \frac{2}{3}}$-norm. We consider the following two subcases.

**Subcase 3.1** $$|\tau + |n|^2| < 2^{-10} |n|^2$$.

In this subcase, we need to evaluate the $\mathcal {F}_{x, t} \big ( \varphi _T \overline{u_{N_1}} \cdot \varphi _T \overline{v_{N_2}} \big )$ term using the $\widehat{X}^{s, \frac{2}{3}}$-norm. By duality and the Cauchy-Schwarz inequality, we have

3.6 $$\begin{aligned} \begin{aligned} \big \Vert&\langle n \rangle ^s \langle \tau + |n|^2 \rangle ^{- \frac{1}{3}} \mathcal {F}_{x, t} \big ( \varphi _T \overline{u_{N_1}} \cdot \varphi _T \overline{v_{N_2}} \big ) \big \Vert _{\ell _n^2 L_\tau ^2 ( \mathfrak {P}_N )} \\&\le N^s \sup _{\Vert h \Vert _{\ell _n^2 L_\tau ^2 ( \mathfrak {P}_N )} \le 1} \bigg | \sum _{\begin{array}{c} n, n_1, n_2 \in \mathbb {Z}^2 \\ n + n_1 + n_2 = 0 \end{array}} \iint _{\tau + \tau _1 + \tau _2 = 0} \widehat{\varphi _T u_{N_1}} (n_1, \tau _1) \widehat{\varphi _T v_{N_2}} (n_2, \tau _2) \\&\qquad \times \frac{h (n, \tau )}{\langle \tau + |n|^2 \rangle ^{\frac{1}{3}}} d\tau d\tau _2 \bigg | \\&\le N^{s} \Vert \widehat{\varphi _T v_{N_2} } \Vert _{\ell _{n_2}^2 L_{\tau _2}^2} \sup _{\Vert h \Vert _{\ell _n^2 L_\tau ^2 ( \mathfrak {P}_N )} \le 1} \bigg \Vert \sum _{\begin{array}{c} n, n_1 \in \mathbb {Z}^2 \\ n + n_1 + n_2 = 0 \end{array}} \int _{\tau + \tau _1 + \tau _2 = 0} \widehat{\varphi _T u_{N_1}} (n_1, \tau _1) \\&\qquad \times \frac{h (n, \tau )}{\langle \tau + |n|^2 \rangle ^{\frac{1}{3}}} d\tau \bigg \Vert _{\ell _{n_2}^2 L_{\tau _2}^2}. \end{aligned} \end{aligned}$$

Let $w_N$ be a space-time distribution that satisfy $\widehat{w_N} (n, \tau ) = h (n, \tau ) / \langle \tau + |n|^2 \rangle ^{\frac{1}{3}}$. Then, using the fact that $\varphi _T$ is supported on $[-1, 1]$ given $0 < T \le \frac{1}{2}$, by the Plancherel theorem, Hölder’s inequality, Lemma 2.4, and Lemma 2.3, we have

$$\begin{aligned} \begin{aligned} \bigg \Vert&\sum _{\begin{array}{c} n, n_1 \in \mathbb {Z}^2 \\ n + n_1 + n_2 = 0 \end{array}} \int _{\tau + \tau _1 + \tau _2 = 0} \widehat{\varphi _T u_{N_1}} (n_1, \tau _1) \frac{h (n, \tau )}{\langle \tau + |n|^2 \rangle ^{\frac{1}{3}}} d\tau \bigg \Vert _{\ell _{n_2}^2 L_{\tau _2}^2} \\&= \Vert \varphi _T u_{N_1} \widetilde{w_N} \Vert _{L_t^2 ([-1, 1]; L_x^2 (\mathbb {T}^2))} \\&\le \Vert \varphi _T u_{N_1} \Vert _{L_t^4 ([-1, 1]; L_x^4 (\mathbb {T}^2))} \Vert w_N \Vert _{L_t^4 ([-1, 1]; L_x^4 (\mathbb {T}^2))} \\&\le N_1^{4\varepsilon } \Vert \varphi _T u_{N_1} \Vert _{X^{0, \frac{1}{2} - \varepsilon }} N^{\frac{1}{3} + \varepsilon } \Vert w_N \Vert _{X^{0, \frac{1}{3}}} \\&\le _\varphi N_1^{-s + 4 \varepsilon } T^{\frac{\varepsilon }{2}} \Vert u_{N_1} \Vert _{X^{s, \frac{2}{3}}} N^{\frac{1}{3} + \varepsilon } \Vert h \Vert _{\ell _n^2 L_\tau ^2 ( \mathfrak {P}_N )}, \end{aligned} \end{aligned}$$

where $\varepsilon > 0$ is arbitrarily small. Thus, continuing with (3.6), we use Lemma 2.3 to obtain

$$\begin{aligned} \big \Vert&\langle n \rangle ^s \langle \tau + |n|^2 \rangle ^{- \frac{1}{3}} \mathcal {F}_{x, t} \big ( \varphi _T \overline{u_{N_1}} \cdot \varphi _T \overline{v_{N_2}} \big ) \big \Vert _{\ell _n^2 L_\tau ^2 ( \mathfrak {P}_N )} \\&\le _\varphi N^{s + \frac{1}{3} + \varepsilon } N_1^{-s + 4 \varepsilon } T^{\frac{\varepsilon }{2}} \Vert u_{N_1} \Vert _{X^{s, \frac{2}{3}}} \big \Vert \widehat{\varphi _T v_{N_2}} \big \Vert _{\ell _{n_2}^2 L_{\tau _2}^2} \\&\le _\varphi N^{s + \frac{1}{3} + \varepsilon } N_1^{-s + 4 \varepsilon } T^\varepsilon \Vert u_{N_1} \Vert _{Z^{s, \frac{2}{3}}} \big \Vert \langle \tau _2 + |n_2|^2 \rangle ^{\frac{\varepsilon }{2}} \widehat{v_{N_2}} \big \Vert _{\ell _{n_2}^2 L_{\tau _2}^2} \\&\le N^{s + \frac{1}{3} + \varepsilon } N_1^{-s + 4 \varepsilon } T^\varepsilon \Vert u_{N_1} \Vert _{Z^{s, \frac{2}{3}}} N_2^{-s - \frac{4}{3} + \varepsilon } \Vert v_{N_2} \Vert _{Y^{s, \frac{2}{3}}} \\&\le N^{s + \frac{1}{3} + \varepsilon } N_1^{-2s - \frac{4}{3} + 5 \varepsilon } T^\varepsilon \Vert u_{N_1} \Vert _{Z^{s, \frac{2}{3}}} \Vert v_{N_2} \Vert _{Z^{s, \frac{2}{3}}}. \end{aligned}$$

Since $s \le -\frac{1}{2}$, we have $s + \frac{1}{3} + \varepsilon < 0$ for $\varepsilon > 0$ small enough. Thus, the above estimate is acceptable if $-2\,s - \frac{4}{3} + 5 \varepsilon \le 0$, which is valid given $s > -\frac{2}{3}$ and $\varepsilon > 0$ sufficiently small.

**Subcase 3.2** $$2^{-10}|n|^2 \le |\tau + |n|^2| < 2^{-10} |n_1|^2$$.

In this subcase, we need to evaluate the $\mathcal {F}_{x, t} \big ( \varphi _T \overline{u_{N_1}} \cdot \varphi _T \overline{v_{N_2}} \big )$ term using the $Y^{s, \frac{2}{3}}$-norm. By duality and the Cauchy-Schwarz inequality, we have

3.7 $$\begin{aligned} \begin{aligned} \big \Vert&\langle \tau + |n|^2 \rangle ^{\frac{s}{2} - \frac{1}{3}} \mathcal {F}_{x, t} \big ( \varphi _T \overline{u_{N_1}} \cdot \varphi _T \overline{v_{N_2}} \big ) \big \Vert _{\ell _n^2 L_\tau ^2 (\mathfrak {P}_N)} \\&\le N^{s + \frac{1}{3}} \sup _{\Vert h \Vert _{\ell _n^2 L_\tau ^2 ( \mathfrak {P}_N )} \le 1} \bigg | \sum _{\begin{array}{c} n, n_1, n_2 \in \mathbb {Z}^2 \\ n + n_1 + n_2 = 0 \end{array}} \iint _{\tau + \tau _1 + \tau _2 = 0} \widehat{\varphi _T u_{N_1}} (n_1, \tau _1) \widehat{\varphi _T v_{N_2}} (n_2, \tau _2) \\&\qquad \times \frac{h (n, \tau )}{\langle \tau + |n|^2 \rangle ^{\frac{1}{2}}} d\tau d\tau _2 \bigg | \\&\le N^{s + \frac{1}{3}} \Vert \widehat{\varphi _T v_{N_2}} \Vert _{\ell _{n_2}^2 L_{\tau _2}^2} \sup _{\Vert h \Vert _{\ell _n^2 L_\tau ^2 ( \mathfrak {P}_N )} \le 1} \bigg \Vert \sum _{\begin{array}{c} n, n_1 \in \mathbb {Z}^2 \\ n + n_1 + n_2 = 0 \end{array}} \int _{\tau + \tau _1 + \tau _2 = 0} \widehat{\varphi _T u_{N_1}} (n_1, \tau _1) \\&\qquad \times \frac{h (n, \tau )}{\langle \tau + |n|^2 \rangle ^{\frac{1}{2}}} d\tau \bigg \Vert _{\ell _{n_2}^2 L_{\tau _2}^2}. \end{aligned} \end{aligned}$$

Note that the first inequality is valid since $s + \frac{1}{3} < 0$ given $s \le - \frac{1}{2}$. Let $w_N$ be a space-time distribution that satisfy $\widehat{w_N} (n, \tau ) = h (n, \tau ) / \langle \tau + |n|^2 \rangle ^{\frac{1}{2}}$. Then, using the fact that $\varphi _T$ is supported on $[-1, 1]$ given $0 < T \le \frac{1}{2}$, by the Plancherel theorem, Hölder’s inequality, Lemma 2.4, and Lemma 2.3, we have

$$\begin{aligned} \bigg \Vert&\sum _{\begin{array}{c} n, n_1 \in \mathbb {Z}^2 \\ n + n_1 + n_2 = 0 \end{array}} \int _{\tau + \tau _1 + \tau _2 = 0} \widehat{\varphi _T u_{N_1}} (n_1, \tau _1) \times \frac{h (n, \tau )}{\langle \tau + |n|^2 \rangle ^{\frac{1}{2}}} d\tau \bigg \Vert _{\ell _{n_2}^2 L_{\tau _2}^2} \\&= \Vert \varphi _T u_{N_1} \widetilde{w_N} \Vert _{L_t^2 ([-1, 1]; L_x^2 (\mathbb {T}^2))} \\&\le \Vert \varphi _T u_{N_1} \Vert _{L_t^4 ([-1, 1]; L_x^4 (\mathbb {T}^2))} \Vert w_N \Vert _{L_t^4 ([-1, 1]; L_x^4 (\mathbb {T}^2))} \\&\le N_1^{4 \varepsilon } \Vert \varphi _T u_{N_1} \Vert _{X^{0, \frac{1}{2} - \varepsilon }} N^\varepsilon \Vert w_N \Vert _{X^{0, \frac{1}{2}}} \\&\le _\varphi N_1^{-s + 4 \varepsilon } T^{\frac{\varepsilon }{2}} \Vert u_{N_1} \Vert _{X^{s, \frac{2}{3}}} N^\varepsilon \Vert h \Vert _{\ell _n^2 L_\tau ^2 (\mathfrak {P}_N)}, \end{aligned}$$

where $\varepsilon > 0$ is arbitrarily small. Thus, continuing with (3.7), we use Lemma 2.3 to obtain

$$\begin{aligned} \big \Vert&\langle \tau + |n|^2 \rangle ^{\frac{s}{2} - \frac{1}{3}} \mathcal {F}_{x, t} \big ( \varphi _T \overline{u_{N_1}} \cdot \varphi _T \overline{v_{N_2}} \big ) \big \Vert _{\ell _n^2 L_\tau ^2 (\mathfrak {P}_N)} \\&\le _\varphi N^{s + \frac{1}{3} + \varepsilon } N_1^{-s + 4 \varepsilon } T^{\frac{\varepsilon }{2}} \Vert u_{N_1} \Vert _{X^{s, \frac{2}{3}}} \big \Vert \widehat{\varphi _T v_{N_2}} \big \Vert _{\ell _{n_2}^2 L_{\tau _2}^2} \\&\le _\varphi N^{s + \frac{1}{3} + \varepsilon } N_1^{-s + 4 \varepsilon } T^\varepsilon \Vert u_{N_1} \Vert _{Z^{s, \frac{2}{3}}} \big \Vert \langle \tau _2 + |n_2|^2 \rangle ^{\frac{\varepsilon }{2}} \widehat{v_{N_2}} \big \Vert _{\ell _{n_2}^2 L_{\tau _2}^2} \\&\le N^{s + \frac{1}{3} + \varepsilon } N_1^{-s + 4 \varepsilon } T^\varepsilon \Vert u_{N_1} \Vert _{Z^{s, \frac{2}{3}}} N_2^{-s - \frac{4}{3} + \varepsilon } \Vert v_{N_2} \Vert _{Y^{s, \frac{2}{3}}} \\&\le N^{s + \frac{1}{3} + \varepsilon } N_1^{-2s - \frac{4}{3} + 5 \varepsilon } T^\varepsilon \Vert u_{N_1} \Vert _{Z^{s, \frac{2}{3}}} \Vert v_{N_2} \Vert _{Z^{s, \frac{2}{3}}}. \end{aligned}$$

Since $s \le -\frac{1}{2}$, we have $s + \frac{1}{3} + \varepsilon < 0$ for $\varepsilon > 0$ small enough. Thus, the above estimate is acceptable if $-2\,s - \frac{4}{3} + 5 \varepsilon \le 0$, which is valid given $s > -\frac{2}{3}$ and $\varepsilon > 0$ sufficiently small.

Also, by the Cauchy-Schwarz inequality, we get

$$\begin{aligned} \big \Vert&\langle n \rangle ^s \langle \tau + |n|^2 \rangle ^{- 1} \mathcal {F}_{x, t} \big ( \varphi _T \overline{u_{N_1}} \cdot \varphi _T \overline{v_{N_2}} \big ) \big \Vert _{\ell _n^2 L_\tau ^1 (\mathfrak {P}_N)} \\&\le \big \Vert \langle n \rangle ^s \langle \tau + |n|^2 \rangle ^{- \frac{1}{2} + \varepsilon } \mathcal {F}_{x, t} \big ( \varphi _T \overline{u_{N_1}} \cdot \varphi _T \overline{v_{N_2}} \big ) \big \Vert _{\ell _n^2 L_\tau ^2 (\mathfrak {P}_N)}, \end{aligned}$$

where $\varepsilon > 0$ is arbitrarily small. The above term can be estimated similarly as above (along with $\langle \tau + |n|^2 \rangle ^{\varepsilon } \le N_1^{2 \varepsilon }$) for the $\ell _n^2 L_\tau ^2$ term. Combining the above two estimates, we obtain the desired inequality.

**Case 4** $$|\tau + |n|^2| < 2^{-10} |n_1|^2$$ and $$|\tau _2 + |n_2|^2| < 2^{-10} |n_2|^2$$.

This case follows similarly from Case 3 by switching the roles of $u_{N_1}$ and $v_{N_2}$. We thus omit details.

Thus, we have finished our proof. $\square$

Before moving on to the proof of our main bilinear estimate in Proposition 3.1, we first observe that by definition of the $X^{s, b}$-norm in (2.1) and the $Y^{s, b}$-norm in (2.3), we have the following decompositions:

$$\begin{aligned} \Vert u \Vert _{X^{s, b}}^2&= \sum _{\begin{array}{c} N \ge 1 \\ \text {dyadic} \end{array}} \Vert u_N \Vert _{X^{s, b}}^2, \\ \Vert u \Vert _{Y^{s, b}}^2&= \sum _{\begin{array}{c} N \ge 1 \\ \text {dyadic} \end{array}} \Vert u_N \Vert _{Y^{s, b}}^2. \end{aligned}$$

Thus, it follows that we have the following decomposition regarding the $Z^{s, b}$ norm:

3.8 $$\begin{aligned} \begin{aligned} \Vert u \Vert _{Z^{s, b}}^2&\sim \Vert P_{\text {lo}} u \Vert _{X^{s, b}}^2 + \Vert P_{\text {hi}} u \Vert _{Y^{s, b}}^2 \\&= \sum _{\begin{array}{c} N \ge 1 \\ \text {dyadic} \end{array}} \big ( \Vert P_{\text {lo}} u_N \Vert _{X^{s, b}}^2 + \Vert P_{\text {hi}} u_N \Vert _{Y^{s, b}}^2 \big ) \\&\sim \sum _{\begin{array}{c} N \ge 1 \\ \text {dyadic} \end{array}} \Vert u_N \Vert _{Z^{s, b}}^2. \end{aligned} \end{aligned}$$

### Proof of Proposition 3.1

By (3.8), we have

3.9 $$\begin{aligned} \begin{aligned} \big \Vert&\langle \tau + |n|^2 \rangle ^{-1} \mathcal {F}_{x, t} \big ( \varphi _T \overline{u} \cdot \varphi _T \overline{v} \big ) (n, \tau ) \big \Vert _{\widehat{Z}^{s, \frac{2}{3}}}^2 \\&\le \sum _{\begin{array}{c} N \ge 1 \\ \text {dyadic} \end{array}} \bigg ( \sum _{\begin{array}{c} N_1, N_2 \ge 1 \\ \text {dyadic} \end{array}} \big \Vert \langle \tau + |n|^2 \rangle ^{-1} \mathcal {F}_{x, t} \big ( \varphi _T \overline{u_{N_1}} \cdot \varphi _T \overline{v_{N_2}} \big ) (n, \tau ) \big \Vert _{\widehat{Z}^{s, \frac{2}{3}} (\mathfrak {P}_N)} \bigg )^2. \end{aligned} \end{aligned}$$

For each nonzero summand on the right-hand side of (3.9), we know that *N*, $N_1$, and $N_2$ must satisfy one of the following:

1. $$2^{-5} N \le N_1 \le 2^5 N$$ and $$N_2 \le 2^6 N$$,
2. $$2^{-5} N \le N_2 \le 2^5 N$$ and $$N_1 \le 2^6 N$$,
3. $$\frac{1}{2} N_1 \le N_2 \le 2N_1$$ and $$N < 2^{-5} N_1$$.

We now treat the above three cases separately.

**Case 1** $$2^{-5} N \le N_1 \le 2^5 N$$ and $$N_2 \le 2^6 N$$.

In this case, by Lemma 3.2, the Cauchy-Schwarz inequality, and (3.8) twice, we have

$$\begin{aligned} (3.9)&\le \sum _{\begin{array}{c} N \ge 1 \\ \text {dyadic} \end{array}} \bigg ( \sum _{\begin{array}{c} N_1 \ge 1 \text { dyadic} \\ 2^{-5} \le N_1/N \le 2^5 \end{array}} \sum _{\begin{array}{c} N_2 \ge 1 \text { dyadic} \\ N_2 \le 2^6 N \end{array}} N_2^{-\delta } T^\theta \Vert u_{N_1} \Vert _{Z^{s, \frac{2}{3}}} \Vert v_{N_2} \Vert _{Z^{s, \frac{2}{3}}} \bigg )^2 \\&\le T^{2 \theta } \sum _{\begin{array}{c} N \ge 1 \\ \text {dyadic} \end{array}} \bigg ( \sum _{\begin{array}{c} N_1 \ge 1 \text { dyadic} \\ 2^{-5} \le N_1/N \le 2^5 \end{array}} \Vert u_{N_1} \Vert _{Z^{s, \frac{2}{3}}} \bigg ( \sum _{\begin{array}{c} N_2 \ge 1 \\ \text {dyadic} \end{array}} \Vert v_{N_2} \Vert _{Z^{s, \frac{2}{3}}}^2 \bigg )^{1/2} \bigg )^2 \\&\le T^{2 \theta } \sum _{\begin{array}{c} N \ge 1 \\ \text {dyadic} \end{array}} \sum _{\begin{array}{c} N_1 \ge 1 \text { dyadic} \\ 2^{-5} \le N_1/N \le 2^5 \end{array}} \Vert u_{N_1} \Vert _{Z^{s, \frac{2}{3}}}^2 \Vert v \Vert _{Z^{s, \frac{2}{3}}}^2 \\&\le T^{2 \theta } \Vert u \Vert _{Z^{s, \frac{2}{3}}}^2 \Vert v \Vert _{Z^{s, \frac{2}{3}}}^2, \end{aligned}$$

where in the right-hand side of the first inequality we have $\delta > 0$.

**Case 2** $$2^{-5} N \le N_2 \le 2^5 N$$ and $$N_1 \le 2^6 N$$.

This case can be treated in the same way as Case 1, and so we omit details.

**Case 3** $$\frac{1}{2} N_1 \le N_2 \le 2N_1$$ and $$N < 2^{-5} N_1$$.

In this case, by Lemma 3.3, the Cauchy-Schwarz inequality, and (3.8) twice, we have

$$\begin{aligned} (3.9)&\le \sum _{\begin{array}{c} N \ge 1 \\ \text {dyadic} \end{array}} \bigg ( \sum _{\begin{array}{c} N_1 \ge 1 \text { dyadic} \\ N_1 > 2^5 N \end{array}} \sum _{\begin{array}{c} N_2 \ge 1 \text { dyadic} \\ 1/2 \le N_2 / N_1 \le 2 \end{array}} N^{-\delta } T^\theta \Vert u_{N_1} \Vert _{Z^{s, \frac{2}{3}}} \Vert v_{N_2} \Vert _{Z^{s, \frac{2}{3}}} \bigg )^2 \\&\le T^{2 \theta } \sum _{\begin{array}{c} N \ge 1 \\ \text {dyadic} \end{array}} N^{-2 \delta } \bigg ( \sum _{\begin{array}{c} N_1 \ge 1 \\ \text {dyadic} \end{array}} \Vert u_{N_1} \Vert _{Z^{s, \frac{2}{3}}}^2 \bigg ) \sum _{\begin{array}{c} N_1 \ge 1 \\ \text {dyadic} \end{array}} \bigg ( \sum _{\begin{array}{c} N_2 \ge 1 \text { dyadic} \\ 1/2 \le N_2 / N_1 \le 2 \end{array}} \Vert v_{N_2} \Vert _{Z^{s, \frac{2}{3}}} \bigg )^2 \\&\le T^{2 \theta } \Vert u \Vert _{Z^{s, \frac{2}{3}}}^2 \sum _{\begin{array}{c} N_1 \ge 1 \\ \text {dyadic} \end{array}} \Vert v_{N_1} \Vert _{Z^{s, \frac{2}{3}}}^2 \\&\le T^{2 \theta } \Vert u \Vert _{Z^{s, \frac{2}{3}}}^2 \Vert v \Vert _{Z^{s, \frac{2}{3}}}^2, \end{aligned}$$

where in the right-hand side of the first inequality we have $\delta > 0$.

Combining the above three cases, we have thus finished our proof. $\square$

## Local Well-Posedness of the Quadratic NLS

In this section, we present the proof of Theorem 1.1, local well-posedness of the quadratic NLS (1.1) in the low regularity setting. As mentioned in Sect. 1, we mainly focus our attention on local well-posedness of (1.1) on $H^s (\mathbb {T}^2)$ for $- \frac{2}{3} < s \le -\frac{1}{2}$, using the estimates of the $Z^{s, b}$-norm in Sects. 2 and 3.

By writing (1.1) in the Duhamel formulation, we have

4.1 $$\begin{aligned} u(t) = \Gamma [u] (t) := e^{it \Delta } u_0 - i \int _0^t e^{i (t - t') \Delta } \overline{u}^2 (t') dt'. \end{aligned}$$

Since we are only interested in local well-posedness, we can insert time cut-off functions. For $0 < T \le \frac{1}{2}$, we let $\eta : \mathbb {R}\rightarrow [0, 1]$ be a smooth function such that $\eta \equiv 1$ on $[-1, 1]$ and $\eta \equiv 0$ outside of $[-2, 2]$ and let $\eta _{2T} (t):= \eta (t / 2T)$. We first replace the two $\overline{u}$’s on the right-hand side of (4.1) by $\eta _{2T} \overline{u}$. Also, note that for any function *F* that is smooth in space and Schwartz in time, we have

$$\begin{aligned} \int _0^t e^{i (t - t') \Delta } F(x, t') dt'&= \int _0^t \sum _{n \in \mathbb {Z}^2} e^{in \cdot x} e^{-i (t - t') |n|^2} \int _\mathbb {R}e^{i t' \tau } \widehat{F} (n, \tau ) d\tau dt' \\&= \sum _{n \in \mathbb {Z}^2} e^{i n \cdot x} \int _\mathbb {R}e^{-it |n|^2} \widehat{F} (n, \tau ) \frac{e^{it (\tau + |n|^2)} - 1}{i (\tau + |n|^2)} d\tau \\&= \sum _{n \in \mathbb {Z}^2} e^{i n \cdot x} \int _\mathbb {R}e^{-it |n|^2} \widehat{F} (n, \tau ) \psi (\tau + |n|^2) \frac{e^{it (\tau + |n|^2)} - 1}{i (\tau + |n|^2)} d\tau \\&\qquad - \sum _{n \in \mathbb {Z}^2} e^{i n \cdot x} \int _\mathbb {R}e^{-it |n|^2} \widehat{F} (n, \tau ) \frac{1 - \psi (\tau + |n|^2)}{i(\tau + |n|^2)} d\tau \\&\qquad + \sum _{n \in \mathbb {Z}^2} e^{i n \cdot x} \int _\mathbb {R}e^{it \tau } \widehat{F} (n, \tau ) \frac{1 - \psi (\tau + |n|^2)}{i(\tau + |n|^2)} d\tau , \end{aligned}$$

where $\psi : \mathbb {R}\rightarrow [0,1]$ is a smooth cut-off function such that $\psi \equiv 1$ on $[-1, 1]$ and $\psi \equiv 0$ outside of $[-2, 2]$. Let us define the following nonlinear terms.

4.2 $$\begin{aligned} \begin{aligned} \mathcal {N}_1 (u, v)&:= -i \eta (t) \sum _{n \in \mathbb {Z}^2} e^{i n \cdot x} \int _\mathbb {R}e^{-it |n|^2} \mathcal {F}_{x, t} \big ( \eta _{2T} \overline{u} \cdot \eta _{2T} \overline{v} \big ) (n, \tau ) \\&\qquad \times \psi (\tau + |n|^2) \frac{e^{it (\tau + |n|^2)} - 1}{i (\tau + |n|^2)} d\tau , \\ \mathcal {N}_2 (u, v)&:= i \eta (t) \sum _{n \in \mathbb {Z}^2} e^{i n \cdot x} \int _\mathbb {R}e^{-it |n|^2} \mathcal {F}_{x, t} \big ( \eta _{2T} \overline{u} \cdot \eta _{2T} \overline{v} \big ) (n, \tau ) \frac{1 - \psi (\tau + |n|^2)}{i(\tau + |n|^2)} d\tau , \\ \mathcal {N}_3 (u, v)&:= -i \sum _{n \in \mathbb {Z}^2} e^{i n \cdot x} \int _\mathbb {R}e^{it \tau } \mathcal {F}_{x, t} \big ( \eta _{2T} \overline{u} \cdot \eta _{2T} \overline{v} \big ) (n, \tau ) \frac{1 - \psi (\tau + |n|^2)}{i(\tau + |n|^2)} d\tau . \end{aligned} \end{aligned}$$

We consider the following formulation of the quadratic NLS (1.1):

4.3 $$\begin{aligned} u(t) = \Gamma _1 [u] (t) := \eta (t) e^{it \Delta } u_0 + \mathcal {N}_1 (u, u) + \mathcal {N}_2 (u, u) + \mathcal {N}_3 (u, u). \end{aligned}$$

### Relevant Estimates

In this subsection, we present some relevant estimates for proving our local well-posedness result. We first show the following homogeneous linear estimate.

#### Lemma 4.1

Let $s \in \mathbb {R}$, $b \in \mathbb {R}$, and $0 < T \le 1$. Then, we have

$$\begin{aligned} \big \Vert \eta (t) e^{it \Delta } \phi \big \Vert _{Z_T^{s, b}} \le _\eta \Vert \phi \Vert _{H^s (\mathbb {T}^2)}, \end{aligned}$$

#### Proof

By the definition of the $Z_T^{s, b}$-norm in (2.6), Lemma 2.7, and Lemma 2.1 with $k = 0$, we have

$$\begin{aligned} \Vert \eta (t) e^{it \Delta } \phi \Vert _{Z_T^{s, b}} \le \Vert \eta (t) e^{it \Delta } \phi \Vert _{Z^{s, b}} \le \Vert \eta (t) e^{it \Delta } \phi \Vert _{X^{s, b}} \le _\eta \Vert \phi \Vert _{H^s (\mathbb {T}^2)}, \end{aligned}$$

as desired. $$\square $$

We now take $b = \frac{2}{3}$ and show the following bilinear estimate.

#### Lemma 4.2

Let $-\frac{2}{3} < s \le -\frac{1}{2}$ and $0 < T \le \frac{1}{4}$. Then, we have

$$\begin{aligned} \Vert \mathcal {N}_1 (u, v) \Vert _{Z_T^{s, \frac{2}{3}}}&\le _\eta T^\theta \Vert u \Vert _{Z_T^{s, \frac{2}{3}}} \Vert v \Vert _{Z_T^{s, \frac{2}{3}}}, \\ \Vert \mathcal {N}_2 (u, v) \Vert _{Z_T^{s, \frac{2}{3}}}&\le _\eta T^\theta \Vert u \Vert _{Z_T^{s, \frac{2}{3}}} \Vert v \Vert _{Z_T^{s, \frac{2}{3}}}, \\ \Vert \mathcal {N}_3 (u, v) \Vert _{Z_T^{s, \frac{2}{3}}}&\le _\eta T^\theta \Vert u \Vert _{Z_T^{s, \frac{2}{3}}} \Vert v \Vert _{Z_T^{s, \frac{2}{3}}} \end{aligned}$$

for some $\theta > 0$, where $\mathcal {N}_1$, $\mathcal {N}_2$, and $\mathcal {N}_3$ are as defined in (4.2).

#### Proof

The idea of the proof comes from [3]. As in the proof of Lemma 2.6, by working with the extensions of *u* and *v* outside $[-T, T]$, it suffices to show the following three estimates:

$$\begin{aligned} \Vert \mathcal {N}_1 (u, v) \Vert _{Z^{s, \frac{2}{3}}}&\le _\eta T^\theta \Vert u \Vert _{Z^{s, \frac{2}{3}}} \Vert v \Vert _{Z^{s, \frac{2}{3}}}, \\ \Vert \mathcal {N}_2 (u, v) \Vert _{Z^{s, \frac{2}{3}}}&\le _\eta T^\theta \Vert u \Vert _{Z^{s, \frac{2}{3}}} \Vert v \Vert _{Z^{s, \frac{2}{3}}}, \\ \Vert \mathcal {N}_3 (u, v) \Vert _{Z^{s, \frac{2}{3}}}&\le _\eta T^\theta \Vert u \Vert _{Z^{s, \frac{2}{3}}} \Vert v \Vert _{Z^{s, \frac{2}{3}}}, \end{aligned}$$

for some $$\theta > 0$$.

To deal with the $\mathcal {N}_1$ term, by Lemma 2.7, the Taylor expansion, Lemma 2.1, Lemma 2.5, and Proposition 3.1, we obtain

$$\begin{aligned} \begin{aligned} \Vert \mathcal {N}_1 (u, v) \Vert _{Z^{s, \frac{2}{3}}}&\le \Vert \mathcal {N}_1 (u, v) \Vert _{X^{s, \frac{2}{3}}} \\&\le \sum _{k = 1}^\infty \frac{1}{k!} \bigg \Vert t^k \eta (t) e^{it \Delta } \sum _{n \in \mathbb {Z}^2} e^{in \cdot x} \int _\mathbb {R}\mathcal {F}_{x, t} \big ( \eta _{2T} \overline{u} \cdot \eta _{2T} \overline{v} \big ) (n, \tau ) \\&\qquad \times \psi (\tau + |n|^2) \langle \tau + |n|^2 \rangle ^{k - 1} d\tau \bigg \Vert _{X^{s, \frac{2}{3}}} \\&\le _\eta \sum _{k = 1}^\infty \frac{3^k}{k!} \bigg \Vert \langle n \rangle ^s \int _\mathbb {R}\mathcal {F}_{x, t} \big ( \eta _{2T} \overline{u} \cdot \eta _{2T} \overline{v} \big ) (n, \tau ) \psi (\tau + |n|^2) \langle \tau + |n|^2 \rangle ^{k - 1} d\tau \bigg \Vert _{\ell _n^2} \\&\le \sum _{k = 1}^\infty \frac{6^k}{k!} \big \Vert \langle n \rangle ^s \langle \tau + |n|^2 \rangle ^{-1} \mathcal {F}_{x, t} \big ( \eta _{2T} \overline{u} \cdot \eta _{2T} \overline{v} \big ) (n, \tau ) \big \Vert _{\ell _n^2 L_\tau ^1} \\&\le \big \Vert \langle \tau + |n|^2 \rangle ^{-1} \mathcal {F}_{x, t} \big ( \eta _{2T} \overline{u} \cdot \eta _{2T} \overline{v} \big ) (n, \tau ) \big \Vert _{\widehat{Z}^{s, \frac{2}{3}}} \\&\le _\eta T^\theta \Vert u \Vert _{Z^{s, \frac{2}{3}}} \Vert v \Vert _{Z^{s, \frac{2}{3}}} \end{aligned} \end{aligned}$$

for some $$\theta > 0$$.

For the $\mathcal {N}_2$ term, using Lemma 2.7, Lemma 2.1 with $k = 0$, the fact that $1 - \psi$ is bounded by 1 and supported outside of $[-1, 1]$, Lemma 2.5, and Proposition 3.1, we have

$$\begin{aligned} \begin{aligned} \Vert \mathcal {N}_2 (u, v) \Vert _{Z^{s, \frac{2}{3}}}&\le \Vert \mathcal {N}_2 (u, v) \Vert _{X^{s, \frac{2}{3}}} \\&\le \bigg \Vert \eta (t) e^{it \Delta } \sum _{n \in \mathbb {Z}^2} e^{in \cdot x} \int _\mathbb {R}\mathcal {F}_{x, t} \big ( \eta _{2T} \overline{u} \cdot \eta _{2T} \overline{v} \big ) (n, \tau ) \\&\qquad \times \frac{1 - \psi (\tau + |n|^2)}{i (\tau + |n|^2)} d\tau \bigg \Vert _{X^{s, \frac{2}{3} }} \\&\le _\eta \bigg \Vert \langle n \rangle ^s \int _\mathbb {R}\mathcal {F}_{x, t} \big ( \eta _{2T} \overline{u} \cdot \eta _{2T} \overline{v} \big ) (n, \tau ) \frac{1 - \psi (\tau + |n|^2)}{i (\tau + |n|^2)} d\tau \bigg \Vert _{\ell _n^2} \\&\le \big \Vert \langle n \rangle ^s \langle \tau + |n|^2 \rangle ^{-1} \mathcal {F}_{x, t} \big ( \eta _{2T} \overline{u} \cdot \eta _{2T} \overline{v} \big ) (n, \tau ) \big \Vert _{\ell _n^2 L_\tau ^1} \\&\le \big \Vert \langle \tau + |n|^2 \rangle ^{-1} \mathcal {F}_{x, t} \big ( \eta _{2T} \overline{u} \cdot \eta _{2T} \overline{v} \big ) (n, \tau ) \big \Vert _{\widehat{Z}^{s, \frac{2}{3}}} \\&\le _\eta T^\theta \Vert u \Vert _{Z^{s, \frac{2}{3}}} \Vert v \Vert _{Z^{s, \frac{2}{3}}} \end{aligned} \end{aligned}$$

for some $$\theta > 0$$.

For the $\mathcal {N}_3$ term, since $1 - \psi$ is bounded by 1 and supported outside of $[-1, 1]$, by the monotonicity property (2.5) and Proposition 3.1, we have

$$\begin{aligned} \begin{aligned} \Vert \mathcal {N}_3 (u, v) \Vert _{Z^{s, \frac{2}{3}}}&= \bigg \Vert \mathcal {F}_{x, t} \big ( \eta _{2T} \overline{u} \cdot \eta _{2T} \overline{v} \big ) (n, \tau ) \frac{1 - \psi (\tau + |n|^2)}{\tau + |n|^2} \bigg \Vert _{\widehat{Z}^{s, \frac{2}{3}}} \\&\le \big \Vert \langle \tau + |n|^2 \rangle ^{-1} \mathcal {F}_{x, t} \big ( \eta _{2T} \overline{u} \cdot \eta _{2T} \overline{v} \big ) \big \Vert _{\widehat{Z}^{s, \frac{2}{3}}} \\&\le _\eta T^\theta \Vert u \Vert _{Z^{s, \frac{2}{3}}} \Vert v \Vert _{Z^{s, \frac{2}{3}}} \end{aligned} \end{aligned}$$

for some $\theta > 0$. Thus, we finish our proof. $\square$

### Local Well-Posedness

We now use the formulation (4.3) and the estimates in Sect. 4.1 to prove our local well-posedness result. We let $0 < T \le \frac{1}{4}$ and fix $-\frac{2}{3} < s \le - \frac{1}{2}$.

For the setting of $\mathbb {T}^2$, by (4.3), Lemma 4.1, and Lemma 4.2, we have

4.4 $$\begin{aligned} \begin{aligned} \big \Vert \Gamma _1 [u] \big \Vert _{Z_T^{s, \frac{2}{3}}}&\le \big \Vert \eta (t) e^{it \Delta } u_0 \big \Vert _{Z_T^{s, \frac{2}{3}}} + \sum _{j = 1}^3 \Vert \mathcal {N}_j (u, u) \Vert _{Z_T^{s, \frac{2}{3}}} \\&\le _\eta \Vert u_0 \Vert _{H^s (\mathbb {T}^2)} + T^\theta \Vert u \Vert _{Z_T^{s, \frac{2}{3}}}^2, \end{aligned} \end{aligned}$$

for some $\theta > 0$. Similarly, we obtain the following difference estimate:

4.5 $$\begin{aligned} \begin{aligned} \big \Vert \Gamma _1 [u] - \Gamma _1 [v] \big \Vert _{Z_T^{s, \frac{2}{3}}}&\le \sum _{j = 1}^3 \Big ( \Vert \mathcal {N}_j (u, u - v) \Vert _{Z_T^{s, \frac{2}{3}}} + \Vert \mathcal {N}_j (u - v, v) \Vert _{Z_T^{s, \frac{2}{3}}} \Big ) \\&\le _\eta T^\theta \Big ( \Vert u \Vert _{Z_T^{s, \frac{2}{3}}} + \Vert v \Vert _{Z_T^{s, \frac{2}{3}}} \Big ) \Vert u - v \Vert _{Z_T^{s, \frac{2}{3}}}. \end{aligned} \end{aligned}$$

Thus, by choosing $T = T(\Vert u_0 \Vert _{H^s (\mathbb {T}^2)}) > 0$ sufficiently small, we have that $\Gamma _1$ is a contraction on the ball $B_R \subset Z^{s, \frac{2}{3}}$ of radius $R \sim \Vert u_0 \Vert _{H^s (\mathbb {T}^2)}$. This gives the existence part of Theorem 1.1 when $\mathcal {M} = \mathbb {T}^2$ and the uniqueness in the ball $B_R$. Also, the continuous dependence of solutions on the initial data follows easily from the formulation (4.3), Lemma 4.1, (4.4), and (4.5).

It remains to extend the uniqueness of solutions to (1.1) to the entire $Z_T^{s, \frac{2}{3}}$-space. We let *u* and *v* be two solutions of (1.1) in $Z_T^{s, \frac{2}{3}}$. Note that *u* and *v* satisfy the formulation (4.3) for $t \in [-T, T]$. For $0 < T_0 \le T$, we use (4.5) to obtain

$$\begin{aligned} \Vert u - v \Vert _{Z_{T_0}^{s, \frac{2}{3}}}&\le _\eta T_0^\theta \Big ( \Vert u \Vert _{Z_{T_0}^{s, \frac{2}{3}}} + \Vert v \Vert _{Z_{T_0}^{s, \frac{2}{3}}} \Big ) \Vert u - v \Vert _{Z_{T_0}^{s, \frac{2}{3}}} \\&\le T_0^\theta \Big ( \Vert u \Vert _{Z_T^{s, \frac{2}{3}}} + \Vert v \Vert _{Z_T^{s, \frac{2}{3}}} \Big ) \Vert u - v \Vert _{Z_{T_0}^{s, \frac{2}{3}}}. \end{aligned}$$

Thus, by choosing

$$\begin{aligned} T_0 = T_0 \Big ( \Vert u \Vert _{Z_T^{s, \frac{2}{3}}}, \Vert v \Vert _{Z_T^{s, \frac{2}{3}}} \Big ) > 0 \end{aligned}$$

sufficiently small, we can use Lemma 2.6 to obtain

$$\begin{aligned} \Vert u - v \Vert _{C ( [-T_0, T_0] ); H^s (\mathbb {T}^2)} \le \Vert u - v \Vert _{Z_{T_0}^{s, \frac{2}{3}}} = 0, \end{aligned}$$

so that $u \equiv v$ on $[-T_0, T_0]$. Since $T_0$ depends only on $\Vert u \Vert _{Z_T^{s, \frac{2}{3}}}$ and $\Vert v \Vert _{Z_T^{s, \frac{2}{3}}}$, we can iterate the above argument on $[-T, -T_0]$ and $[T_0, T]$. This shows that $u \equiv v$ on $[-T, T]$ after a finite number of iterations, and so the uniqueness of (1.1) on the entire $Z_T^{s, \frac{2}{3}}$-space follows.

## Data Availability

Not applicable.

## Footnotes

1 Here, $\frac{1}{2} +$ means $\frac{1}{2} + \varepsilon$ for some $\varepsilon > 0$.

2 On $\mathbb {T}^d$, this framework works only for local well-posedness for small initial data. See Remark 1.3 for a discussion of local well-posedness on periodic domains for large initial data.
